# Impact of Inoculating with Indigenous *Bacillus marcorestinctum* YC-1 on Quality and Microbial Communities of Yibin Yacai (Fermented Mustard) during the Fermentation Process

**DOI:** 10.3390/foods11223593

**Published:** 2022-11-11

**Authors:** Yixin Zhong, Yuke Zou, Zimeng Zheng, Qian Chen, Wei Xu, Yanping Wu, Jia Gao, Kai Zhong, Hong Gao

**Affiliations:** 1 College of Biomass Science and Engineering, Sichuan University, Chengdu 610065, China; 2 The Agricultural and Rural Bureau of Lu County, Luzhou 646100, China; 3 Institute of Agro-Products Processing Science and Technology, Sichuan Academy of Agricultural Sciences, Chengdu 610066, China

**Keywords:** Yibin Yacai, *Bacillus marcorestinctum* YC-1, inoculation, metabolites, microbiome

## Abstract

*Bacillus* species play an important role in improving the quality of some fermented foods and are also one of the dominant bacteria in Yibin Yacai (fermented mustard). However, little is known about their effects on the quality of Yibin Yacai. Here, the effect of *Bacillus marcorestinctum* YC-1 on the quality and microbial communities of Yibin Yacai during the fermentation process was investigated. Results indicated that the inoculation of *Bacillus marcorestinctum* YC-1 promoted the growth of *Weissella* spp. and *Lactobacillus* spp. and inhibited the growth of pathogens, accelerating the synthesis of free amino acids and organic acids and the degradation of nitrite. Furthermore, inoculating Yibin Yacai with YC-1 could effectively enhance the synthesis of alcohols and terpenoids in yeasts, thus producing more linalool, terpinen-4-ol, and α-muurolen in Yibin Yacai, and endowing it with pleasant floral, fruity, woody, and spicy aromas. These findings reveal that the inoculation of *B. marcorestinctum* YC-1 can improve the quality and safety of Yibin Yacai by changing microbial communities as fermentation proceeds.

## 1. Introduction

Fermented foods have received increasing attention because of their nutritional and health benefits [1]. Among these foods, fermented vegetables are widely favored by people thanks to their rich nutrients and flavors and prolonged shelf-life [2]. In China, Yibin Yacai, one of the most typical fermented vegetables, has received increasing attention due to its favorable fragrance, crispness, and sweetness; its annual processing is up to 200,000 tons [3,4].

Yibin Yacai is traditionally manufactured by spontaneous fermentation and contains a wide variety of microbial communities during fermentation and storage [5]. In Yibin Yacai, *Halomonas* and *Bacillus* were found as the dominant bacteria, while *Saccharomycetales* and *Debaryomyces* were the major fungi [6,7]. Microbes play a crucial role in the unique flavor formation of fermented vegetables; thus, the inoculation of a pure strain as a starter becomes one of the main methods to improve the quality of fermented vegetables [8,9]. Some microbial communities are positively correlated with the good quality of fermented products [10]. For instance, *Bacillus* spp. can positively affect the flavor formation of soy sauce [11]. The participation of *Bacillus licheniformis* can shorten the fermentation period and enrich the metabolite profile, thus improving the functionality and safety of sufu [12]. Moreover, bioaugmentation inoculation of *Bacillus* spp. could increase the abundance of *Lactobacillus* and *Candida*, which were considered the core microbes in Daqu, and thus improve the flavor character of Daqu [13,14]. Our previous studies also demonstrated that the inoculation of *Bacillus* spp. could promote the growth of *Lactobacillus* and *Lactococcus*, enhance flavor, and improve the safety of Sichuan paocai [15,16]. However, the correlation between quality and microbial communities during the fermentation of Yibin Yacai is largely unknown. We surmised that the inoculation of *Bacillus* spp. could positively tune the fermentation of microbial communities involved in Yibin Yacai. Hence, systematic research on the correlation between quality and microbial communities during the fermentation period is performed to elucidate the properties of Yibin Yacai.

To this end, herein, *B. marcorestinctum* YC-1 (NCBI GenBank accession No.: OM 033504; Figure S1A,B and Table S1), a *Bacillus* spp. isolated from a commercial Yibin Yacai in our lab [17], was inoculated as a starter to ferment Yibin Yacai in this study, and its role in changing the physicochemical characteristics, flavor-relevant compounds, and microbial communities of the resulting Yibin Yacai during fermentation were systematically investigated. Meanwhile, the correlation between metabolites and microbes after *B. marcorestinctum* YC-1 inoculation was established to reveal its positive role in adjusting the fermentation of Yibin Yacai. The obtained results provide great insights into how to inoculate *Bacillus* spp. to tune the fermentation of Yibin Yacai in order to produce high-quality, safe Yibin Yacai.

## 2. Materials and Methods

### 2.1. Preparation and Sampling of Yibin Yacai

*B. marcorestinctum* YC-1 was isolated from a commercial Yibin Yacai with a fermentation of 5 years (Sichuan Hefeisi Biotechnology Co., LTD., Sichuan, China). The safety evaluation showed that *B. marcorestinctum* YC-1 exhibits no hemolytic activity (Figure S1C), and no resistance to the tested antibiotics (Table S2), demonstrating great potential in fermenting food. *B. marcorestinctum* YC-1 was cultured on a nutrient agar solid medium (NA) at 37 °C for 24 h. Then, a single colony was inoculated into nutrient broth medium (NB) and shaken for 24 h until the concentration of bacterial solution reached 10^8^ CFUs/mL. The starter of *B. marcorestinctum* YC-1 was collected by centrifugation at 6000 rpm for 10 min at 4 °C.

**Figure 1:**
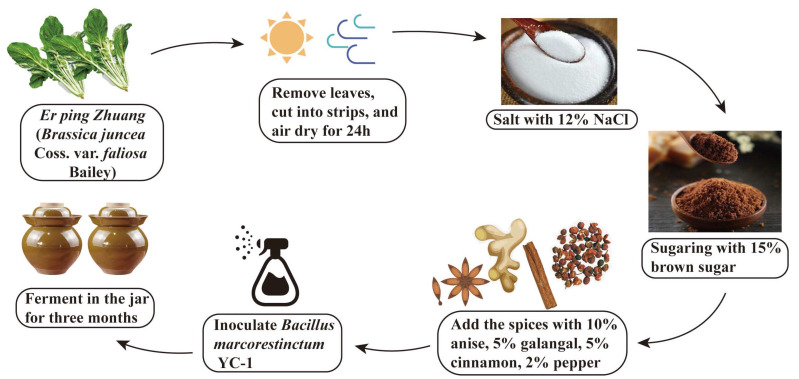
The main fermentation processes of Yibin Yacai inoculated with *Bacillus marcorestinctum* YC-1, based on the traditional fermentation processes.

The fresh *Er ping Zhuang (Brassica juncea* Coss. var. *faliosa* Bailey, belonging to the Cruciferae family), were collected locally in Yibin city, and its manufacturing process is detailed in Figure 1. Firstly, the separated roots were cut into even strips and ventilated for 24 h. After salting with 12% NaCl for 24 h, Yacai was obtained by washing with warm water. Then, Yacai was sugared with 15% brown sugar for 24 h, which was further seasoned with spices, including 10% anise, 5% galangal, 5% cinnamon, and 2% Sichuan pepper. To investigate the role of *B. marcorestinctum* YC-1 inoculation, two groups were fermented in glass jars at 20–25 °C for three months to obtain Yibin Yacai. One group called BMF was inoculated with *B. marcorestinctum* YC-1, while the other one, NF, was untreated. After 10, 30, 60, and 90 days of fermentation, BMF and NF were sampled in triplicate and stored at −80 °C for the subsequent test.

### 2.2. Determination of Physicochemical Characteristics

The pH, reducing sugar, nitrite, and salinity of both NF and BMF samples after 10, 30, 60, and 90 days of fermentation were determined. Reducing sugar and nitrite were measured according to Chinese national standards (GB 5009.7-2016 and GB 5009.33-2016). pH of the samples was measured on a pH meter (PHS25, INESA, Shanghai, China), and their salinity was determined by a salinity meter (ES-421, ATAGO, Tokyo, Japan).

### 2.3. Metabolite Analysis

#### 2.3.1. Organic Acids (OAs) Analysis

The content of OAs was measured by high-performance liquid chromatography (HPLC) according to a published procedure [18]. The separation was carried out on an Amethyst C18–H column (5 μm, 4.6 × 250 mm, Sepax Technologies, Inc., Newark, DE, USA) at a temperature of 30 °C, and CH_3_OH/H_2_O (5:95, *v*/*v*) was used as an eluent. The flow rate of HPLC 1260 Infinity Ⅱ (Agilent Technologies, Inc, Palo Alto, CA, USA) was 0.6 mL/min, and the injection volume was 20 μL. OAs were detected by a diode array detector (DAD) at 210 nm.

#### 2.3.2. Free Amino Acids (FAAs) Analysis

The extraction of FAAs was according to the Chinese national standard (GB/T 30987-2020) and analyzed by an Automatic amino acid analyzer A300 (MembraPure GmbH, Berlin, Germany), as suggested by a reported reference [16]. The injection volume was 20 μL, and the chromatogram was analyzed by aminoPeak software.

#### 2.3.3. Volatile Compounds (VCs) Analysis

VCs in Yibin Yacai were collected by head space solid phase microextraction (HS-SPME), according to a modified procedure [19]. The SPME holder 57330-U (Supelco, Sigma Aldrich, St. Louis, MO, USA) with fiber (DVB/CAR/PDMS, 50/30 μm) was used for the collection of VCs, and the obtained VCs were detected and isolated by a gas chromatography-mass spectrometer (GCMS-QP2010 SE, Shimadzu, Kyoto, Japan). The temperature program of GC-MS was suggested by a previous study [17], and the VCs were identified by calculating their retention indices (RI) based on n-alkanes (C8–C19) according to NIST14s MS data library. 3-Octanol was used as an internal standard to quantify the identified VCs, and the specific odors of identified VCs were analyzed on Perflavory (http://www.perflavory.com/, last accessed on 29 September 2022).

### 2.4. Microbes Analysis

#### 2.4.1. DNA Extraction

Total genomic DNA was extracted using a DNA Extraction Kit (QIAGEN, Dusseldorf, Germany), and the concentration of DNA was verified on agarose gels with NanoDrop 2000 (Thermo Fisher, Waltham, MA, USA). Using genomic DNA as the template, bar code primers and Tks Gflex DNA polymerase (R060B, Takara, Kyoto, Japan) were used for PCR amplification. V3–V4 variable regions of 16S rRNA genes were amplified with universal primers 343F (5′-TACGGRAGGCAGCAG-3′) and 798R (5′-AGGGTATCTAATCCT-3′) for the analysis of bacterial diversity, while ITS I variable regions were amplified with universal primers ITS1F (5′-CTTGGTCATTTAGA GGAAGTAA-3′) and ITS2R (5′-GCTGCGTTCTTCATCGATGC-3′) for the analysis of fungal diversity. The purified amplicons were quantified by Qubit dsDNA assay kit (Q32854, Life Technologies, Carlsbad, CA, USA), and sequenced on an Illumina MiSeq platform (Illumina, San Diego, CA, USA) of OE biotech (Shanghai OE Biotechnology Co., Ltd., Shanghai, China).

#### 2.4.2. Bioinformatic Analysis

Trimmomatic software was used for intercepting the fuzzy base (N) of reads, and retaining the previous high quality sequence when the average base quality was below 20 [20]. The reads with ambiguous, homologous sequences or below 200 bp were abandoned, and only the reads with 75% of bases above Q20 were selected through QIIME software (Version 1.8.0) [21]. After that, the clean reads were subjected to primer sequences removal and clustered to generate operational taxonomic units (OTUs) using Vsearch software (Version 2.4.2) [22] with a 97% similarity cut-off. The representative read of each OTU was selected using the QIIME package and annotated and blasted according to the database.

### 2.5. Statistical Analysis

All experiments were conducted in triplicate, and the results were represented as mean ± standard deviation. The significant differences (*p* < 0.05) were measured by One-way ANOVA and Student’s *T*-test in IBM SPSS Statistics 26 software (SPSS Inc., Chicago, IL, USA). The graphs were generated from the Origin Software 2021 (OriginLab Corporation, Hampton, MA, USA), and orthogonal partial least squares-discriminant analysis (OPLS-DA) and principal coordinates analysis (PCoA) were carried out using SIMCA-P software (Umetrics, Umea, Sweden). The metabolic pathway was analyzed based on the Kyoto Encyclopedia of Genes and Genomes (KEGG) pathway database (https://www.genome.jp/kegg/pathway.html, last accessed on 29 September 2022), and the correlation was analyzed by R Package (AT&T BellLaboratories, Murihale, NJ, USA).

## 3. Results and Discussion

### 3.1. Physicochemical Characteristics

pH plays an important role in fermentation, and a value of about 4 usually indicates the maturity of fermented vegetables [5,23]. As shown in Table 1, the pH value of both groups reached 4 at the end of fermentation, revealing the maturity of Yibin Yacai. However, BMF had lower total acid value of 11.81 g/kg compared with NF (13.81 g/kg), suggesting that inoculation of YC-1 decreased the production of acids during fermentation.

Reducing sugar, as a kind of carbon source, can be metabolized and converted to flavors by microbes [24]. In the present study, the reducing sugar contents of both two groups kept increasing throughout the fermentation (Table 1). On day 90, the reducing sugar reached 11.31 g/100 g in BMF, which was significantly higher than that in NF (9.83 g/100 g). This finding might be associated with the activities of microbes with cellulase secretion, because reducing sugar can be mainly obtained from cellulose in vegetables by the action of cellulase secreted by microbes [10].

Nitrite is a vital index to evaluate the safety of fermented foods and can be formed from nitrate by nitrate reductase [24]. On day 90, the nitrite content was 2.37 mg/kg in BMF, which was only 46% of that in NF at the same time (Table 1). This was consistent with the results of physiological and biochemical identification tests (Table S1), which suggested that the inoculation of *B. marcorestinctum* YC-1 could promote the nitrite metabolic pathway [16]. Salinity can directly affect the taste and flavor of fermented foods by changing microbial structure [25]. During fermentation, the salinity of all samples remained between 3% and 4%; it was lowest on day 60, which was 2.97% in NF and 3.08% in BMF. The results showed that fermentation had little effect on the salinity during the fermentation of Yibin Yacai. Collectively, the inoculation of *B. marcorestinctum* YC-1 could effectively increase the content of reducing sugar and decrease the content of nitrite, thereby generating Yibin Yacai with enhanced nutritional value and safety.

### 3.2. Metabolic Changes during Fermentation

#### 3.2.1. Changes in OAs during Fermentation

**Figure 2:**
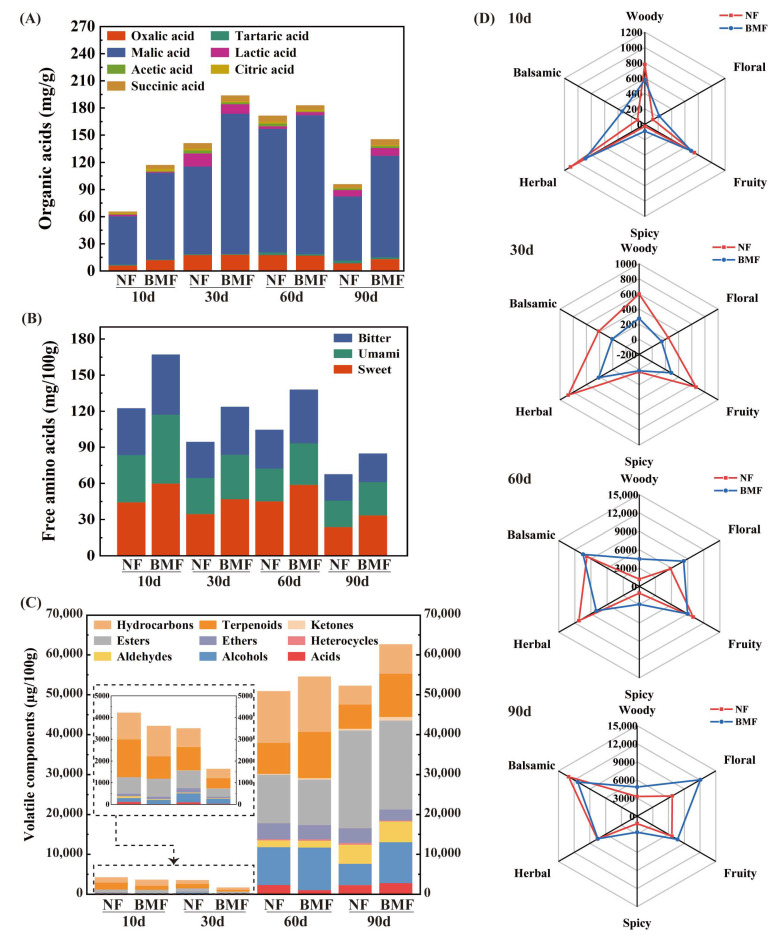
Changes of metabolites in NF and BMF on days 10, 30, 60, and 90 of fermentation. (**A**) Changes in OAs contents. (**B**) Changes in FAAs contents. (**C**) Changes in VCs contents. (**D**) Aroma profiles of NF and BMF based on the odorants of VCs.

As important metabolites of microbes, OAs, not only provide unique flavors to Yibin Yacai, but also inhibit the growth of undesirable microbes [26]. As shown in Figure 2A, seven OAs were detected, and their contents in both groups increased initially, then declined after 30 days of fermentation. Compared to NF, BMF had a higher content of OAs after 90 days of fermentation. Its OA value reached 145.39 mg/g on day 90, which was 1.52 times higher than that of NF. This higher OA content represented that Yibin Yacai in BMF groups possessed a unique, mixed, pleasant odor, for OAs can endow fermented foods with distinctive odors, such as pungent, sour, vinegar-like, and cheesy [27].

Among these 7 OAs, malic acid was the most abundant during the whole fermentation process, which accounted for 72% and 77% of the total OAs in NF and BMF, respectively, on day 90. These results were in accordance with a previous study that found malic acid is the major organic acid in cruciferous vegetables [2]. In addition, the contents of lactic and acetic acids in both groups were increased after 90 days of fermentation, which could be attributed to the lactic fermentation involved in the fermentation process [2,28]. At the end of fermentation, BMF had a higher content of lactic and acetic acids than NF, suggesting that inoculation with *B. marcorestinctum* YC-1 may promote lactic fermentation by changing microbial communities [23]. The above results revealed that the inoculation of *B. marcorestinctum* YC-1 can significantly increase the contents of OAs, promoting fermentation and tuning flavors of Yibin Yacai.

#### 3.2.2. Changes in FAAs during Fermentation

Seventeen FAAs were detected in all samples (Table S4) and can be divided into sweet amino acids, umami amino acids, and bitter amino acids [29]. As shown in Figure 2B, BMF always had a higher content of FAAs than NF, even though the contents of FAAs mostly declined during the entire fermentation. On day 90, BMF contained 84.72 mg/100 g of FAAs, while NF contained 62.27 mg/100 g (Table S4). It is worth noting that sweet amino acids were the major FAAs, and BMF on day 90 had 33.48 mg/100 g of sweet amino acids, which was higher than NF (23.88 mg/100 g) (Table S4).

FAAs are mainly converted from proteins by the decomposition of peptidase, and the richer contents of FAAs in BMF might be due to the promotion of peptidase activity after inoculation [30]. However, the contents of FAAs decreased as the fermentation progressed, because they could be converted into small flavoring molecules by microbes [9]. Additionally, FAAs are important contributors to the taste [29], and sweet and umami amino acids were significantly increased, thus enhancing the taste of sweetness and freshness in BMF. Overall, the inoculation of *B. marcorestinctum* YC-1 could significantly increase the contents of FAAs in Yibin Yacai, enriching its final sweet and umami flavors.

#### 3.2.3. Changes in VCs during Fermentation

A total of 126 VCs were detected in NF and BMF (Tables S5 and S6), and they were grouped as acids, alcohols, aldehydes, heterocycles, ethers, esters, ketones, terpenoids, and hydrocarbons according to their chemical structures (Figure 2C). In NF and BMF, the contents of VCs showed a decline in the early stage, and then significantly increased until the end of fermentation. On day 90, BMF presented 62,617.96 µg/100 g of VCs, while NF had 52,200.85 µg/100 g, and esters and terpenoids were the major VCs in both groups (Tables S5 and S6). Furthermore, a significant increase of terpenoids was found in both groups after fermentation, and 10,885.64 µg/100 g of terpenoids was detected in BMF on day 90, which was 1.79 times higher than that in NF (6066.98 µg/100 g). Interestingly, methyl cinnamate, ethyl cinnamate, (+)-α-pinene, and γ-elemene, were only found in BMF, and these esters and terpenoids contributed to balsamic, sweet, fruity, and spicy aromas. Meanwhile, a significant high content of 10,270.92 µg/100 g for alcohols was found in BMF on day 90, which was 92% higher than that in NF. Among these alcohols, terpinen-4-ol and γ-terpineol only existed in BMF, which contained the fragrance of lilac and pine. Furthermore, the content of linalool was significantly increased in BMF on day 90 (4355.95 µg/100 g), about 2.13 times higher than that in NF (2046.01 µg/100 g), which could confer Yibin Yacai with sweet, floral, and fruity-like citrus aromas [24].

Based on these identified VCs, six representative odorants were selected according to a previous study [31], and characteristic odorant analysis was performed. As depicted in Figure 2D, a great difference was shown between the two groups at the end of fermentation. Floral and fruity were dominant aromas in BMF, and balsamic and herbal were dominant aromas in NF. These findings were consistent with the results of VCs analyses. Combined with the results of VCs and characteristic odorant analyses, we can conclude that BMF presented a better flavor than NF at the end of fermentation, suggesting that the inoculation of *B. marcorestinctum* YC-1 could greatly improve the richness of VCs in Yibin Yacai, generating a better flavor.

### 3.3. Significant Metabolites

**Figure 3:**
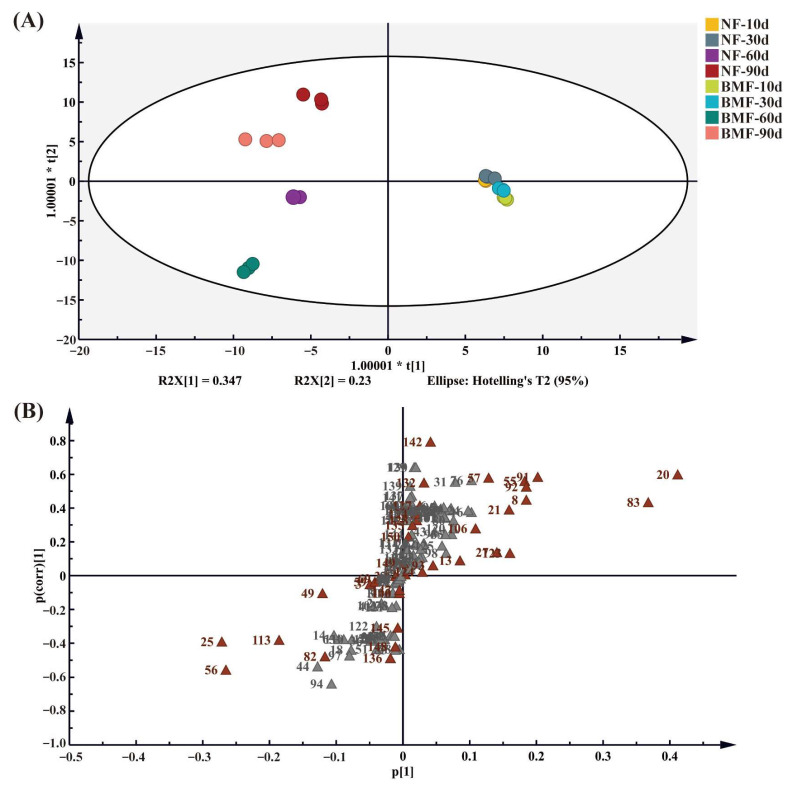
The analysis of dynamics changes of metabolites in NF and BMF during fermentation. (**A**) The OPLS-DA plot based on metabolites, including OAs, FAAs and VCs on days 10, 30, 60, and 90 of fermentation in NF and BMF. (**B**) The S-plot based on the metabolites in NF and BMF.

To figure out the difference in metabolites between NF and BMF, OPLS-DA and S-plot models were performed (Figure 3). As shown in Figure 3A, NF and BMF can be completely separated on days 60 and 90, while the spatial distance was not far on day 30, indicating that fermentation after day 30 was an important period for flavor development of Yibin Yacai. Furthermore, variable importance in projection (VIP) was measured in OPLS-DA, and S-plot was constructed to identify the metabolites contributing to the discrimination based on VIP. As shown in Figure 3B and Table S7, 31 metabolites were screened out as differential metabolites (VIP > 1 and *p* < 0.05), including 8 FAAs, 3 OAs, and 20 VCs (Table S8). These results clearly revealed that VCs, including alcohols, esters, and terpenoids, were the main differential metabolites that contributed to the flavor of Yibin Yacai.

### 3.4. Diversity of Microbial Communities in Yibin Yacai

A total of 6580 OTUs in bacteria and 4153 OTUs in fungi were identified, and the result of α-diversity analysis is shown in Figure S2. The Chao1 index presents the richness of community, while Shannon and Simpson indices present the evenness of community [15]. As depicted in Figure S2A, except for the constant decrease of the Chao 1 index in NF, the three indexes of bacteria in both groups continued to drop until day 60. The Chao1 index in BMF was lower than that in NF, which might be explained by the inoculation of *B. marcorestinctum* YC-1 changing the microbial structure, resulting in an increase in *Bacillus* richness and a decrease in community richness. In fungi, the Chao1 index declined as fermentation progressed, and Shannon and Simpson indices increased firstly, and then significantly decreased on day 90 (Figure S2B). The α-diversity indices of fungi in BMF were richer than that in NF. Therefore, the inoculation of *B. marcorestinctum* YC-1 significantly affected the composition of both bacterial and fungal communities in Yibin Yacai.

### 3.5. Microbial Profile in Yibin Yacai

**Figure 4:**
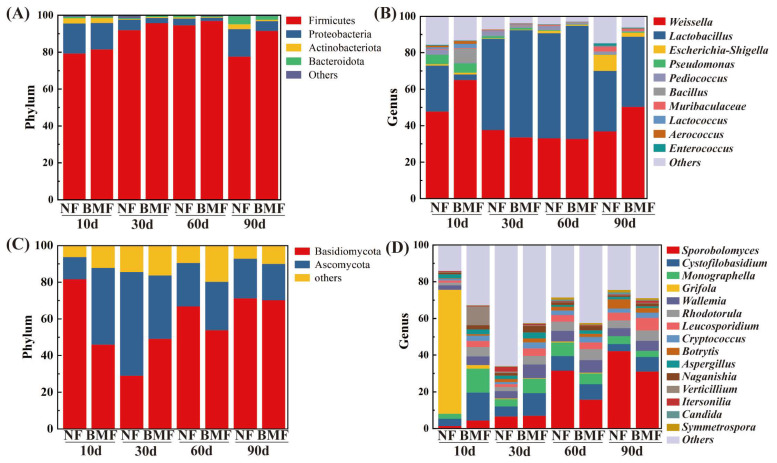
The comparison of relative abundance of microbes on days 10, 30, 60, and 90 of fermentation in NF and BMF. (**A**) Relative abundance (%) of bacteria at the phylum level. (**B**) Relative abundance (%) of bacteria at the genus level. (**C**) Relative abundance (%) of fungi at the phylum level. (**D**) Relative abundance (%) of fungi at the genus level.

At the phylum and genus level, microbes with a relative abundance > 1% were shown in Figure 4. Firmicutes and Proteobacteria were two major bacterial phyla in the tested samples, which were also found in other fermented vegetables [5,9]. The relative abundance of Firmicutes reached its highest on day 60, which was 97.01% in BMF and 94.68% in NF (Figure 4A), while the highest relative abundance of Proteobacteria was 16.30% in NF and 14.43% in BMF on day 10. At the genus level, *Weissella* and *Lactobacillus* were the dominant genera, while *Pseudomonas*, *Escherichia-Shigella*, and *Pediococcus* were the second dominant genera (Figure 4B), and they were commonly found in fermented vegetables [2,15].

On day 10, the relative abundance of *Lactobacillus* was 3.15% in BMF, much less than that of NF (25.23%), while the number of *Lactobacillus* significantly grew on day 30. On the other hand, *Bacillus* (7.68%) significantly increased in BMF on day 10 and decreased on day 30. *Lactobacillus* is an important bacterium for its tolerance to the anaerobic and high salt environment, and it can degrade sugar to produce acids [10,27]. These results indicated that the natural growth of *Lactobacillus* might be negatively affected by the participation of external *B. marcorestinctum* YC-1 in the initial fermentation stage, resulting in less production of acids in BMF. In addition, BMF had more *Weissella* (50.46%) and *Lactobacillus* (38.44%) on day 90 than NF (37.03% and 33.16%), and these two bacteria can produce antimicrobial agents to inhibit the growth of pernicious microbes [25,32]. Compared with NF, a less relative abundance of *Escherichia coli* existed in BMF on day 90 (Figure S3A) because of the increase of *Weissella* and *Lactobacillus*. In consequence, the inoculation of *B. marcorestinctum* YC-1 promoted the growth of LAB, thereby improving the safety of Yibin Yacai.

With regard to fungi, Basidiomycota and Ascomycota were the main phyla (Figure 4C), and the difference between NF and BMF mainly existed on days 10 and 30. In comparison to NF, the relative abundance of Ascomycota in BMF increased by 29.76% on day 10, and the relative abundance of Basidiomycota increased by 20.08% on day 30. At the genus level, *Sporobolomyces*, *Cystofilobasidium*, and *Monographella* were major fungi (Figure 4D), and they were reported as the characteristic fungi in the mustard varieties [5,33]. After inoculation, BMF had more *Monographella* (13.12% and 7.89%) than NF (2.79% and 3.95%) on days 10 and 30. Besides, the relative abundance of *Cystofilobasidium* (7.85–15.18%) in BMF remained higher than that in NF (3.92–7.96%) through the entire fermentation. These above-mentioned fungi have been proven to release a variety of metabolism-related enzymes associated with the synthesis of flavors [34,35]. In addition, *Cystofilobasidium macerans* existed during the whole fermentation (Figure S3B), which can produce extracellular enzymes with high proteolytic and cellulose hydrolysis activity [36], thus facilitating the generation of reducing sugar in Yibin Yacai. Therefore, the inoculation of *B. marcorestinctum* YC-1 favored the growth of fungi that can produce metabolism-related hydrolases, which was conducive to the production Yibin Yacai with better flavor.

### 3.6. Significant Microbes and Predicted Functions of Bacteria

**Figure 5:**
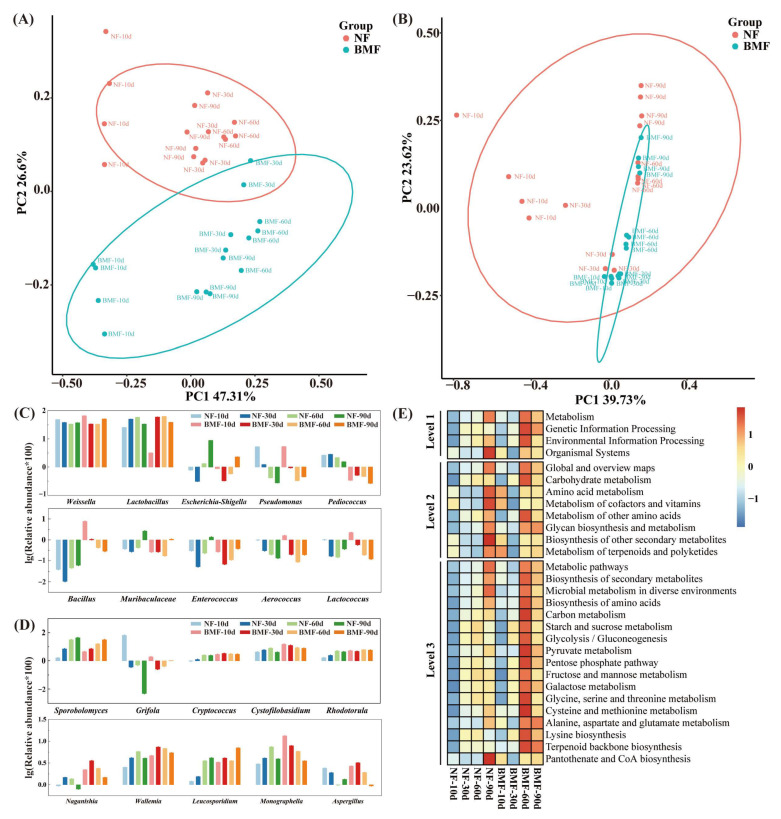
The analysis of dynamic changes in microbial communities between NF and BMF on days 10, 30, 60, and 90 of fermentation. (**A**) PCoA analysis of the bacterial communities based on the Bray-Curtis distance. (**B**) PCoA analysis of the fungal communities based on the Bray-Curtis distance. (**C**) Boxplots of differential bacteria with their abundance between NF and BMF. (**D**) Boxplots of differential fungi with their abundance between NF and BMF. (**E**) The heatmap of predictive functions of bacterial community in NF and BMF.

PCoA analysis based on Bray-Curtis distance was conducted to explore the microbial community differences between the two groups. The variances of PC1 and PC2 were 47.31% and 26.6%, respectively, in bacteria (Figure 5A), and the variances of PC1 and PC2 were 39.73% and 23.62%, respectively, in fungi (Figure 5B). BMF and NF differed greatly in bacterial community for being almost separated, and the position of NF mainly changed in PC1, while the changes of BMF were shown both in PC1 and PC2. As for fungi, BMF showed smaller changes in spatial position compared with NF, and the difference mainly existed on days 10 and 60. These findings proved that the inoculation of *B. marcorestinctum* YC-1 significantly changed the composition of microbial community in Yibin Yacai.

Additionally, the top 10 differential bacteria and fungi at the genus level were analyzed, and the details are shown in Figure 5C,D. *Weissella*, *Lactobacillus*, *Pediococcus, Bacillus, Aerococcus*, and *Lactococcus* were the main differential bacteria, while the difference of *Escherichia-Shigella*, *Pseudomonas*, *Muribaculaceae*, and *Enterococcus* between the two groups was observed in the late stage of fermentation (60–90 days). On the other hand, *Sporobolomyces*, *Grifola*, *Cystofilobasidium*, *Naganishia*, *Wallemia*, *Leucosporidium*, and *Aspergillus* were the significant differential fungi.

The biological functions of differential bacteria in NF and BMF were predicted by PICRUSt, and evaluated by the relative abundance in KEGG pathways (Figure 5E). As a result, the significant difference was mainly in the late fermentation stage (60–90 days), and the proportion of metabolism-related difference was the most in KEGG level-1 pathway analysis, suggesting that the function of differential bacteria was mainly related to metabolism. In KEGG level-2, compared with NF, the relative abundance of amino acid metabolism, and metabolism of other amino acids, significantly increased in BMF on day 60, and the relative abundance in the metabolism of terpenoids and polyketides was higher on days 10 and 60. At level 3 of KEGG classification, BMF had a richer relative abundance in the biosynthesis of amino acids and terpenoids backbone on days 60 and 90 than NF. Notably, a boost in FAAs, terpenoids, glycolysis/gluconeogenesis, and pyruvate metabolisms on days 60 and 90 was only observed in BMF, and these metabolisms were vital pathways to produce flavors (Xiao et al., 2021). Collectively, the inoculation of *B. marcorestinctum* YC-1 significantly increased microbial metabolisms, thus greatly facilitating the production of flavors.

### 3.7. Correlation between Differential Microbes and Metabolites

**Figure 6:**
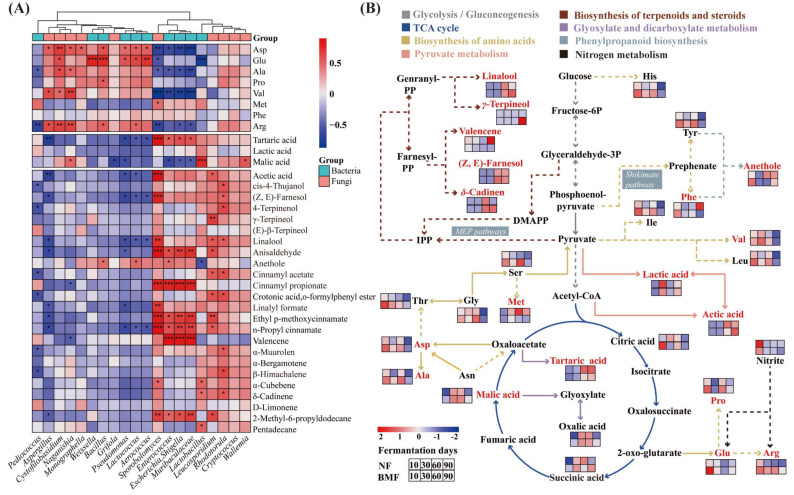
(**A**) The correlation of differential metabolites and differential microbes. (**B**) Metabolic pathway map of microbes according to KEGG and heatmap reflecting the changes of some major metabolites. The solid lines indicate direct synthesis, while the dotted lines indicate indirect synthesis. *: *p* < 0.1, **: *p* < 0.01, ***: *p* < 0.001.

Pearson correlation analysis was performed to reveal the correlation between microbes and metabolites (Figure 6A). The related metabolic pathways were depicted to systematically investigate the influence of inoculation of *B. marcorestinctum* YC-1 on Yibin Yacai (Figure 6B). The correlation analysis revealed that *Cystofilobasidium*, *Bacillus*, and *Lactococcus* were positively related to Asp, Glu, and Pro (Figure 6A), and their relative abundances were increased in BMF compared to NF, which was in accordance with the higher contents of Asp, Glu, and Pro (Figure 2B and Table S4). Moreover, *Weissella*, as the dominant bacteria during the entire fermentation (Figure 4B), showed a positive correlation with umami and sweet amino acids, consistent with previous observations [16]. In particular, brown sugar was added during the manufacture of Yibin Yacai, and sucrose is its main ingredient, which is preferentially favored by microbes as carbon source [8], thus generating FAAs through the tricarboxylic acid cycle under aminopeptidase and transaminase secreted by microbes [30].

Lactic acid, one of the essential OAs in fermented vegetables, not only provides a unique flavor, but also increases the acidity of fermented vegetables [9]. Pyruvate is an important precursor of OAs, which can be converted into lactic acid and other OAs under the catalysis of pyruvate dehydrogenase, pyruvate oxidase, and acetokinase [27]. Moreover, *Lactobacillus* was reported as a main producer of enzymes associated with pyruvate conversion [8], which showed a positive correlation with lactic acid and malic acid in this study (Figure 6A). Compared to NF, BMF presented a higher relative abundance of *Lactobacillus* during fermentation (Figure 4B), seen in the fact that the dominant role of *Lactobacillus* in the synthesis of OAs had promoted after inoculation with *B. marcorestinctum* YC-1, which was consistent with higher content of OAs in BMF (Figure 2A and Table S3).

Nitrite can be converted into ammonia when catalyzed by nitrate reductase and nitrite reductase (Figure 6B), and ammonia can be consumed by *Lactobacillus* as a nitrogen source to produce Glu and Arg [16]. Furthermore, malic acid and tartaric acid have been reported to facilitate the degradation of nitrite, because they can also act as nutrients to support the growth of *Lactobacillus* [37]. Therefore, a significantly higher content of OAs and lower content of nitrite in BMF (Table 1 and Table S3) were associated with a higher abundance of *Lactobacillus* (Figure 4B), suggesting that the inoculation promoted the activity of *Lactobacillus*, thus accelerating the degradation of nitrite and indirectly increasing the FAAs content.

VCs can create favorable aromas in fermented vegetables, and glucose metabolism is a vital pathway for the formation of VCs [30]. As shown in Figure 2A and Figure 6B, terpenoids, as the major VCs in Yibin Yacai, are derived from isopentenyl diphosphate (IPP) and dimethyl diphosphate (DMAPP). IPP and DMAPP can be obtained by the methylerythritol 4-phosphate (MEP) pathway from pyruvate and glyceraldehyde-3phosphate, then metabolized into more complex terpenoids (Figure 6B) [38]. Notably, terpinen-4-ol, crotonic acid, o-formylphenyl ester, and β-himachalene were only found in BMF (Table S5), and they had a significant positive relationship with *Rhodotorula* (Figure 6A). Moreover, linalool, cinnamyl acetate, and α-muurolen contents were higher in BMF and showed a positive relationship with *Leucosporidium* and *Rhodotorula* (Figure 6A). These results suggested that the unique VCs in Yibin Yacai of BMF were mainly influenced by yeasts, such as *Rhodotorula*, *Leucosporidium*, *Cryptococcus*, and *Wallemia*.

Salting Yibin Yacai with 12% NaCl at the beginning could make salt-tolerant yeasts (*Sporobolomyces*, *Cystofilobasidium*, *Wallemia*, and *Rhodotorula*) the core fungi during fermentation. These yeasts produce more stable hydrolases that not only directly utilize reducing sugar to create flavors, but also secrete glycosidase to hydrolyze glycosides, further generating terpenoids [8,30,39]. Additionally, yeasts can metabolize symbiotically with LAB, and assimilate other compounds to produce carbon sources for LAB [35], suggesting that yeasts and LAB had the same positive correlation to VCs in Yibin Yacai. For instance, *Sporobolomyces*, *Leucosporidium*, and *Enterococcus*, three representative LABs, all had a significant positive relationship with production of anisaldehyde and ethyl p-methoxycinnamate (Figure 6A). Both *Lactobacillus* and *Sporobolomyces* were positively correlated with α-cubebene (Figure 6A). Furthermore, LAB can embellish the flavors produced by yeasts during malolactic fermentation [38], which may also account for the richer aromas in BMF.

## 4. Conclusions

In the present study, the effect of *B. marcorestinctum* YC-1 as a starter on the quality of Yibin Yacai was investigated. The results showed that the quality of Yibin Yacai was significantly improved after inoculation. In particular, the abundance of LAB (*Weissella, Lactobacillus*) and yeasts had a significant increase through inoculation, thus resulting in more FAAs, OAs, terpenoids and alcohols generated, endowing strong fruity, floral, and sweet flavors, and accelerating the degradation of nitrite in Yibin Yacai. The change in the microbial community during fermentation after inoculation revealed the strong correlation between metabolites and microbes. Furthermore, we found that yeasts played a more prominent role in the synthesis of terpenoids and alcohols, contributing desirable flavor profiles. Overall, the inoculation of *B. marcorestinctum* YC-1 enriched the flavors, promoted safety, and further improved the quality of Yibin Yacai. These results provide a new direction for the application of *Bacillus* spp. in fermented vegetables.

## Figures and Tables

**Table 1:** The physicochemical characteristics of NF and BMF at different fermentation times.

Days	pH		Total Acids (g/kg)		Reducing Sugar (g/100 g)		Nitrite (mg/kg)		Salinity (%)	
	NF	BMF	NF	BMF	NF	BMF	NF	BMF	NF	BMF
0	5.38	5.38	1.39 ± 0.01	1.39 ± 0.01	3.39 ± 0.19	3.39 ± 0.19	18.98 ± 0.44	18.98 ± 0.44	6.25	6.25
10	4.85	5.12	3.77 ± 0.70	2.92 ± 0.41	8.64 ± 0.06	9.87 ± 0.37 *	17.08 ± 1.77	11.41 ± 0.83 *	ND	3.90
30	4.04	4.22	14.07 ± 0.27	9.62 ± 0.57 *	8.82 ± 1.14	10.46 ± 0.25 *	4.34 ± 0.19	5.15 ± 0.21 *	3.18	3.52 *
60	4.00	4.10	16.61 ± 0.05	13.40 ± 0.31 *	9.40 ± 0.19	11.02 ± 0.12 *	5.12 ± 0.31	4.83 ± 13.40 *	2.97	3.08
90	4.02	4.00	13.81 ± 0.22	11.81 ± 0.11 *	9.83 ± 0.41	11.31 ± 0.07 *	5.14 ± 0.14	2.37 ± 0.07 *	3.32	3.75 *

Data are shown as means ± standard deviation (*n* = 3). Means with * within a column are significantly different compared with NF (*p* < 0.05). ND means not detected.

## Data Availability

Data is contained within the article or supplementary material.

## Supplementary Materials

The following supporting information can be downloaded at: https://www.mdpi.com/article/10.3390/foods11223593/s1, Figure S1: (A) Scanning electron microscopy (SEM) of *Bacillus marcorestinctum* YC-1. (B) Neighbor-joining phylogenetic tree of *Bacillus marcorestinctum* YC-1 (C) The result of hemolytic activity in *Bacillus marcorestinctum* YC-1. The left is a positive of *Bacillus cereus* ATCC 14579, and hemolysis is shown in the white dotted box; Figure S2: The α-diversity analysis of microbial communities in NF and BMF on days 10, 30, 60, 90 of fermentation. (A) Bacteria. (B) Fungi. Figure S3: The microbial communities’ composition at species level in NF and BMF on days 10, 30, 60, 90 of fermentation. (A) Bacteria. (B) Fungi. Table S1: Physiological and biochemical identification results; Table S2: The results of the susceptibility test; Table S3: The contents of OAs in NF and BMF during fermentation; Table S4: The contents of FAAs in NF and BMF during fermentation; Table S5: Detailed information and contents of main VCs in NF; Table S6: Detailed information and contents of main VCs in BMF; Table S7: Ordinal list of metabolites in S-plot; Table S8: Differential metabolites between NF and BMF.

## References

[1] Mukherjee A., Gomez-Sala B., O’Connor E.M., Kenny J.G., Cotter P.D. (2022). Global Regulatory Frameworks for Fermented Foods: A Review. Front Nutr..

[2] Xiao Y., Xiong T., Peng Z., Liu C., Huang T., Yu H., Xie M. (2018). Correlation between microbiota and flavours in fermentation of Chinese Sichuan Paocai. Food Res. Int..

[3] Liu D., Wu M., Xiao L. (2021). Processing status and developing technological path of pickled sprouts. Chin. Condiment.

[4] He C., Jin X., Zeng X. (2022). Present situation and suggestion of Yibin Yacai industry development. Mod. Agric. Technol..

[5] Zhang C., Zhang J., Liu D. (2021). Biochemical changes and microbial community dynamics during spontaneous fermentation of Zhacai, a traditional pickled mustard tuber from China. Int. J. Food Microbiol..

[6] Zuo Y., Wang X., Ye B. (2016). Based on high-throughput sequencing analysis of bacterial community structure in Yibin sprouts. Sci. Technol. Food Ind..

[7] Bai G., Zou W., Li H. (2019). Analysis of microbial diversity of the finished product of sweet sprouts by high-throughput sequencing technology. Chin. Condiment.

[8] Xiao M., Huang T., Xu Y., Peng Z., Liu Z., Guan Q., Xie M., Xiong T. (2021). Metatranscriptomics reveals the gene functions and metabolic properties of the major microbial community during Chinese Sichuan Paocai fermentation. Food Microbiol..

[9] Yang Y., Fan Y., Li T., Yang Y., Zeng F., Wang H., Suo H., Song J., Zhang Y. (2022). Microbial composition and correlation between microbiota and quality-related physiochemical characteristics in chongqing radish paocai. Food Chem..

[10] Zhang F., Tang Y., Ren Y., Yao K., He Q., Wan Y., Chi Y. (2018). Microbial composition of spoiled industrial-scale Sichuan paocai and characteristics of the microorganisms responsible for paocai spoilage. Int. J. Food Microbiol..

[11] Zhao G., Liu C., Hadiatullah H., Yao Y., Lu F. (2021). Effect of Hericium erinaceus on bacterial diversity and volatile flavor changes of soy sauce. LWT Food Sci. Technol..

[12] Liu J., Chen J., Li s., Tian w., Wu H., Han B. (2021). Comparison of volatile and non-volatile metabolites in sufu produced with bacillus licheniformis by rapid fermentation. Int. J. Food Prop..

[13] Yang Y., Zou Y., Zeng K., Chen D., Li Z., Guo H., Huang D., Wang X., Luo H. (2022). Effect of *Bacillus subtilis* fortified inoculation on the microbial communities in different niches of Daqu. J. Biosci. Bioeng..

[14] He G., Dong Y., Huang J., Wang X., Zhang S., Wu C., Jin Y., Zhou R. (2019). Alteration of microbial community for improving flavor character of Daqu by inoculation with Bacillus velezensis and Bacillus subtilis. LWT.

[15] Yang Z., Luo F., Zhong K., Huang C., Yu Z., Peng Z., Wu Y., Bu Q., Gao H. (2021). Effect of *Bacillus subtilis* Y61 inoculation on bacterial community and metabolic profile of sichuan paocai fermentation. LWT Food Sci. Technol..

[16] Luo F., Yang Z., Zhong K., Huang C., Yu Z., Peng Z., Wu Y., Bu Q., Gao H. (2021). Effects of *Bacillus megaterium* L222 on quality and bacterial diversity of Sichuan paocai. Food Res. Int..

[17] Zou Y., Zhong Y., Huang L., Xu W., Wu Y., Gao J., Zhong K., Gao H. (2022). Effects of brown sugar addition and fermentation time on metabolites and microbial communities of Yibin Yacai. LWT Food Sci. Technol..

[18] Yang J., Zuo Y., Ma Y. (2020). Changes of main components and their correlation with the changes of nitrite content in Yibin sprouts during the salt-adding fermentation. Food Ferment. Ind..

[19] Zhang J., Deng J., Shi J., Changqing Z., Xingxiu Z., Peng W., Hongying Z. (2014). Principal components analysis of volatile aroma components of five kinds of Yibin sprouts. Sci. Technol. Food Ind..

[20] Bolger A.M., Lohse M., Usadel B. (2014). Trimmomatic: A flexible trimmer for Illumina sequence data. Bioinformatics.

[21] Caporaso J.G., Kuczynski J., Stombaugh J., Bittinger K. (2010). QIIME allows analysis of high-throughput community sequencing data. Nat. Methods.

[22] Rognes T., Flouri T., Nichols B., Quince C., Mahe F. (2016). VSEARCH: A versatile open source tool for metagenomics. PeerJ.

[23] Liang H., He Z., Wang X., Song G., Huiying C., Xinping L., Chaofan J., Shengjie L. (2020). Effects of salt concentration on microbial diversity and volatile compounds during suancai fermentation. Food Microbiol..

[24] Huang Y., Jia X., Yu J., Chen Y., Liu D., Liang M. (2021). Effect of different lactic acid bacteria on nitrite degradation, volatile profiles, and sensory quality in Chinese traditional paocai. LWT Food Sci. Technol..

[25] Yang X., Hu W., Jiang A., Xiu Z. (2019). Effect of salt concentration on quality of Chinese northeast sauerkraut fermented by *Leuconostoc mesenteroides* and *Lactobacillus plantarum*. Food Biosci..

[26] Valledor S.J.D., Dioso C.M., Bucheli JE V., Park Y.J., Suh D.H., Jung E.S., Kim B., Holzapfel W.H., Todorov S.D. (2022). Characterization and safety evaluation of two beneficial, enterocin-producing *Enterococcus faecium* strains isolated from kimchi, a Korean fermented cabbage. Food Microbiol..

[27] Zhao N., Zhang C., Yang Q., Yang B., Lu W., Li D., Liu X., Tian F., Zhang H., Chen W. (2016). Multiple roles of lactic acid bacteria microflora in the formation of marker flavour compounds in traditional Chinese paocai. RSC Adv..

[28] Rao Y., Tao Y., Chen X., She X., Qian Y., Li Y., Du Y., Xiang W., Li H., Liu L. (2020). The characteristics and correlation of the microbial communities and flavors in traditionally pickled radishes. LWT Food Sci. Technol..

[29] Delompre T., Guichard E., Briand L., Salles C. (2019). Taste Perception of Nutrients Found in Nutritional Supplements: A Review. Nutrients.

[30] Ye Z., Shang Z., Li M., Zhang X., Ren H., Hu X., Yi J. (2022). Effect of ripening and variety on the physiochemical quality and flavor of fermented Chinese chili pepper (Paojiao). Food Chem..

[31] Zhao Y., Wei W., Tang L., Wang D., Wang Y., Wu Z., Zhang W. (2021). Characterization of aroma and bacteria profiles of Sichuan industrial paocai by HS-SPME-GC-O-MS and 16S rRNA amplicon sequencing. Food Res. Int..

[32] Liu X., Qu H., Gou M., Guo H., Wang L., Yan X. (2020). Application of *Weissella cibaria* X31 or *Weissella confusa* L2 as a starter in low nitrite dry-fermented sausages. Int. J. Food Eng..

[33] Wu J., Zeng R., Zhang J. (2020). Fungal Diversity of Pickled Kohlrabi during Fermentation Analyzed by High-throughput Sequencing. Food Sci..

[34] Przemieniecki S.W., Kurowski T.P., Damszel M., Krawczyk K., Karwowska A. (2018). Effectiveness of the *Bacillus* sp SP-A9 Strain as a Biological Control Agent for Spring Wheat (*Triticum aestivum* L.). J. Agric. Sci. Technol..

[35] Kot A.M., Kieliszek M., Piwowarek K., Błażejak S., Mussagy C.U. (2021). Sporobolomyces and Sporidiobolus—Non-conventional yeasts for use in industries. Fungal Biol. Rev..

[36] Chreptowicz K., Mierzejewska J., Tkacova J., Mlynek M., Certik M. (2019). Carotenoid-producing yeasts: Identification and Characteristics of Environmental Isolates with a Valuable Extracellular Enzymatic Activity. Microorganisms.

[37] Xu K., Cheng L., Me Y., Qiao C., Zeng F. (2019). Changes in organic acids and effects on nitrite degradation during pickled cowpea (Vigna sinensis) fermentation. Food Ferment. Ind..

[38] Mele M.A., Kang H.M., Lee Y.T., Islam M.Z. (2021). Grape terpenoids: Flavor importance, genetic regulation, and future potential. Crit. Rev. Food Sci. Nutr..

[39] Chen K., Liu C., Wang Y., Wang Z., Li F., Ma L., Li J. (2021). Predominance of indigenous non-*Saccharomyces* yeasts in the traditional fermentation of greengage wine and their significant contribution to the evolution of terpenes and ethyl esters. Food Res. Int..

