# Supplementary material for: Impact of Inoculating with Indigenous Bacillus marcorestinctum YC-1 on Quality and Microbial Communities of Yibin Yacai (Fermented Mustard) during the Fermentation Process
Source: Foods. 2022 Nov 11;11(22):3593. doi: 10.3390/foods11223593 (PMC9689668; doi:10.3390/foods11223593)
Supplement: Supplementary file 1 [file foods-11-03593-s001.zip › foods-1970455-supplementary.pdf]

Supplementary Materials

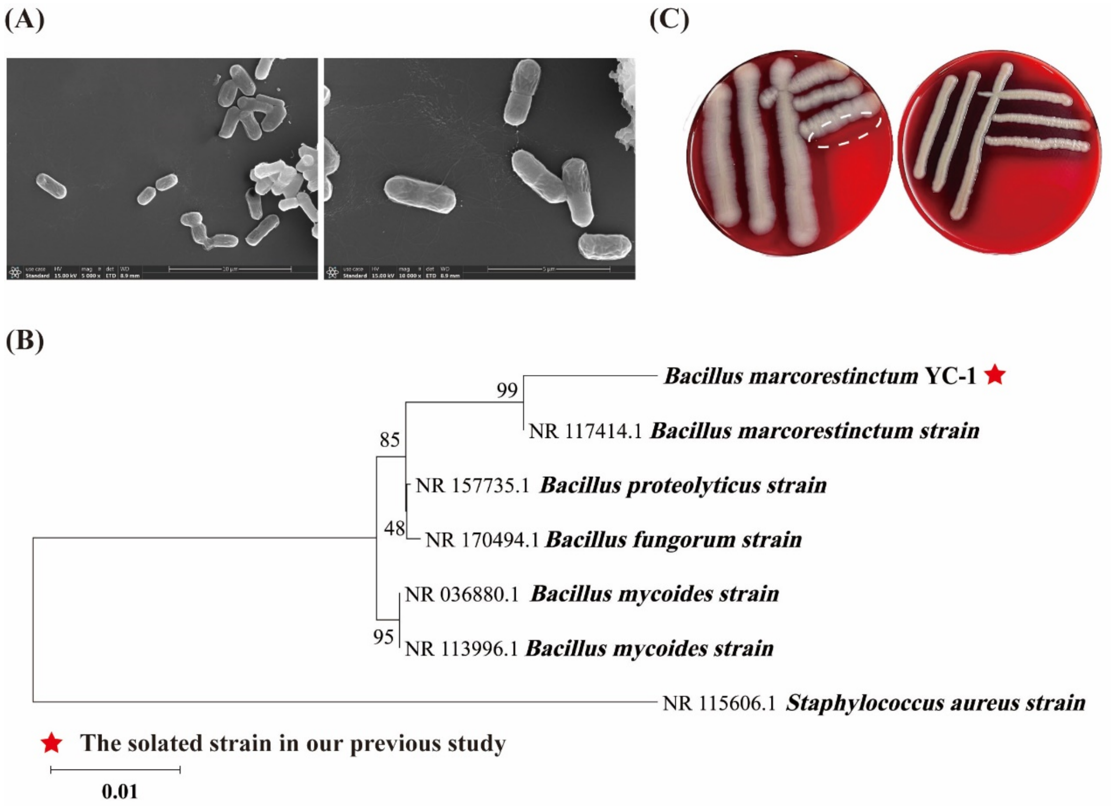

Figure. S1 (A) Scanning electron microscopy (SEM) of *Bacillus marcorestinctum* YC-1. (B) Neighbor-joining phylogenetic tree of *Bacillus marcorestinctum* YC-1 (C) The results of hemolytic activity in *Bacillus marcorestinctum* YC-1. The left was a positive of *Bacillus cereus* ATCC 14579, and hemolysis was shown in the white dotted box.

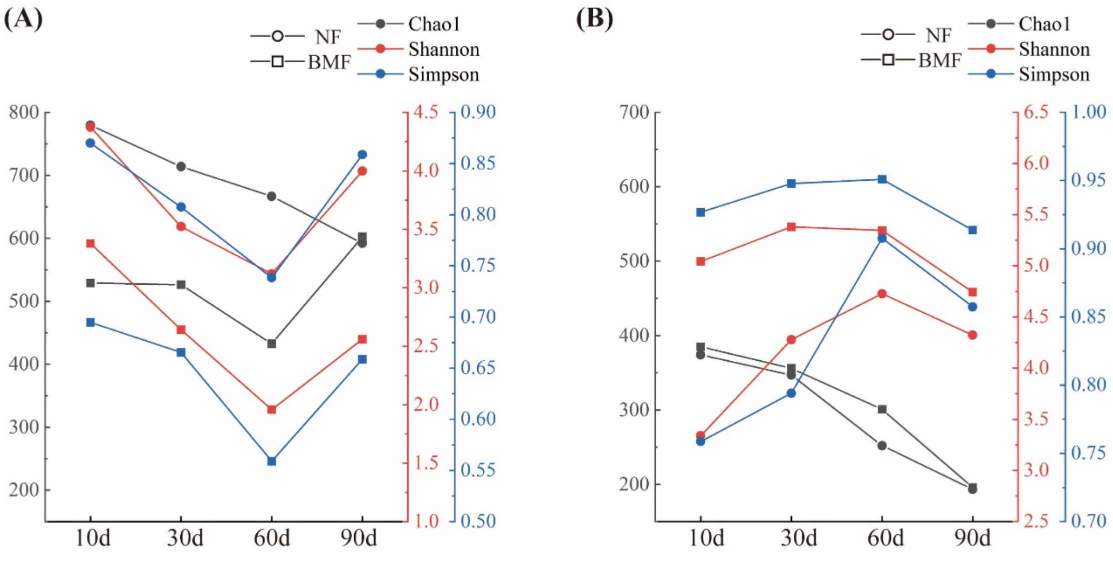

Figure S2: The α-diversity analysis of microbial communities in NF and BMF on days 10, 30, 60, 90 of fermentation. (A) Bacteria. (B) Fungi.

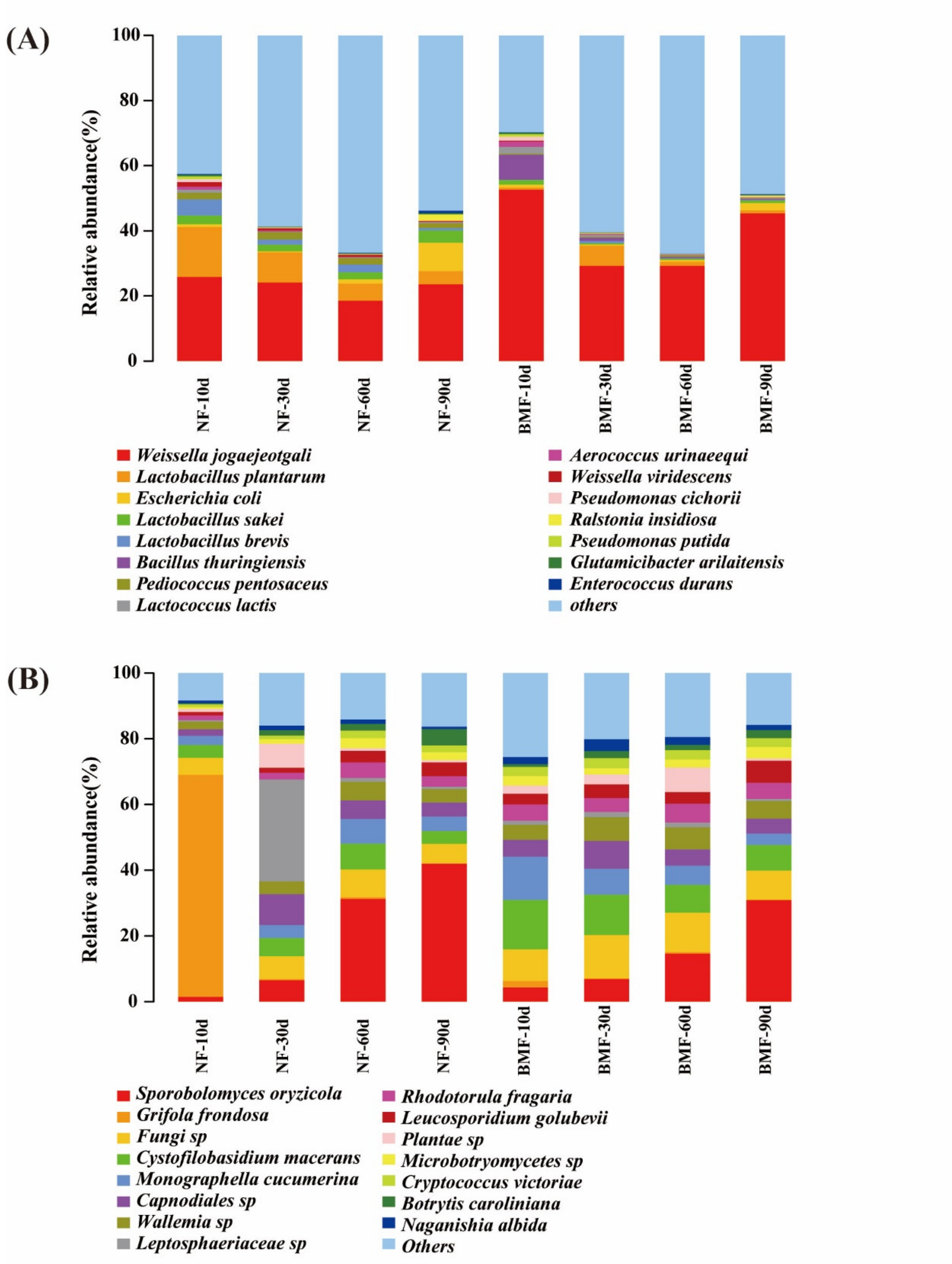

(A)
Relative abundance(%)
0
20
40
60
80
100
NF-10d
NF-30d
NF-60d
NF-90d
BMF-10d
BMF-30d
BMF-60d
BMF-90d
Weissella jogaejeotgali
Lactobacillus plantarum
Escherichia coli
Lactobacillus sakei
Lactobacillus brevis
Bacillus thuringiensis
Pediococcus pentosaceus
Lactococcus lactis
Aerococcus urinaeequi
Weissella viridescens
Pseudomonas cichorii
Ralstonia insidiosa
Pseudomonas putida
Glutamicibacter arilaitensis
Enterococcus durans
others
(B)
Relative abundance(%)
0
20
40
60
80
100
NF-10d
NF-30d
NF-60d
NF-90d
BMF-10d
BMF-30d
BMF-60d
BMF-90d
Sporobolomyces oryzicola
Grifola frondosa
Fungi sp
Cystofilobasidium macerans
Monographella cucumerina
Capnodiales sp
Wallemia sp
Leptosphaeriaceae sp
Rhodotorula fragaria
Leucosporidium golubevii
Plantae sp
Microbotryomycetes sp
Cryptococcus victoriae
Botrytis caroliniana
Naganishia albida
Others

Figure S3: The microbial communities' composition at species level in NF and BMF on days 10, 30, 60, 90 of fermentation. (A) Bacteria. (B) Fungi.

Table S1. Physiological and biochemical identification results

**Substrate**	**Reaction**
Peptone	N
Sucrose	N
Nitrate	P
Cellobiose	N
Glucose (aerogenesis)	P
Glucose	P
Galactose	N
Arabinose	N
Glycerin	N
Hydrogen sulfide	N
Melezitose	N
Rhamnose	N

P means positive, and N means negative

Table S2. The results of susceptibility test

	Antibiotics	Sensitivity
E	Erythromycin	I
CC	Clindamycin	S
IPM	imipenem	S
CIP	Ciprofloxacin	S
C	Chloramphenicol	S
GM	Gentamicin	S
VA	Vancomycin	S
TE	Tetracycline	S

I means intermediary, and S means sensitive.

Table S3. The contents of OAs in NF and BMF during fermentation

OAs	The contents of OAs (mg/g)							
	**NF-10D**	**BMF-10D**	**NF-30D**	**BMF-30D**	**NF-60D**	**BMF-60D**	**NF-90D**	**BMF-90D**
Oxalic acid	6.39 ± 0.08	12.22 ± 0.28 *	17.96 ± 0.17	18.02 ± 0.11	17.88 ± 0.41	17.24 ± 0.18	8.73 ± 0.18	13.35 ± 0.12 *
Tartaric acid	0.70 ± 0.29	0.54 ± 0.00	1.02 ± 0.66	0.93 ± 0.37	2.91 ± 0.67	1.79 ± 0.21	2.95 ± 0.09	2.02 ± 0.21 *
Malic acid	53.66 ± 1.11	95.91 ± 3.41 *	145.15 ± 2.08	154.91 ± 2.38 *	136.61 ± 1.90	153.43 ± 1.55 *	70.93 ± 0.58	112.20 ± 0.56 *
Lactic acid	1.74 ± 0.50	1.24 ± 0.27	14.47 ± 2.08	10.67 ± 0.43 *	2.68 ± 0.57	3.57 ± 0.73	7.05 ± 0.61	8.46 ± 0.67 *
Acetic acid	0.57 ± 0.43	0.50 ± 0.35	3.26 ± 0.83	1.86 ± 0.09 *	3.26 ± 0.89	0.64 ± 0.03 *	1.68 ± 0.30	2.18 ± 0.39
Citric acid	1.46 ± 0.04	2.67 ± 0.64 *	2.04 ± 0.43	1.25 ± 0.22 *	1.79 ± 0.07	1.91 ± 0.80	1.05 ± 0.10	1.16 ± 0.02
Succinic acid	0.87 ± 0.02	3.74 ± 0.69 *	5.29 ± 0.69	5.92 ± 0.40	6.26 ± 0.34	4.22 ± 0.11 *	3.16 ± 0.53	6.03 ± 1.88
In total	65.40	116.82	189.18	193.57	171.38	182.80	95.55	145.39

Data are shown as means ± standard deviation (n = 3).

Means with * within a column are significantly different compared with NF ($p < 0.05$).

Table S4. The contents of FAAs in NF and BMF during fermentation

FAAs	The contents of FAAs (mg/100g)							
	**NF-10D**	**BMF-10D**	**NF-30D**	**BMF-30D**	**NF-60D**	**BMF-60D**	**NF-90D**	**BMF-90D**
Sweet amino acids								
Thr	4.54 ± 0.53	6.33 ± 0.04 *	4.11 ± 0.05	5.29 ± 0.14 *	4.82 ± 0.06	6.14 ± 0.10 *	2.87 ± 0.03	3.93 ± 0.08 *
Ser	4.02 ± 0.16	5.36 ± 0.04 *	3.25 ± 0.02	4.16 ± 0.22 *	3.66 ± 0.06	4.80 ± 0.07 *	2.15 ± 0.02	2.95 ± 0.09 *
Gly	3.24 ± 0.24	3.86 ± 0.25 *	3.60 ± 1.52	3.23 ± 0.38	3.59 ± 0.32	4.50 ± 0.22 *	1.38 ± 0.04	2.02 ± 0.07 *
Ala	16.50 ± 0.24	21.17 ± 0.49 *	12.60 ± 0.00	16.57 ± 0.61 *	16.16 ± 0.43	22.01 ± 0.29 *	7.52 ± 0.06	11.52 ± 0.33 *
Lys	4.98 ± 0.06	6.36 ± 0.27 *	4.05 ± 0.04	5.32 ± 0.12	4.94 ± 0.21	6.00 ± 0.14 *	2.46 ± 0.07	3.64 ± 0.06 *
Pro	11.22 ± 0.15	16.98 ± 0.35 *	10.04 ± 0.13	12.49 ± 0.52 *	12.03 ± 0.67	15.45 ± 0.30 *	7.49 ± 0.02	9.42 ± 0.22 *
In total	44.51	60.07	37.65	47.07	45.20	58.90	23.88	33.48
Umami amino acids								
Asp	26.34 ± 0.23	32.25 ± 0.27 *	19.59 ± 0.24	24.87 ± 0.35 *	24.65 ± 0.51	28.43 ± 0.14 *	13.41 ± 0.03	16.99 ± 0.38 *
Glu	12.84 ± 0.27	25.03 ± 5.50 *	10.43 ± 5.04	12.09 ± 1.41	2.62 ± 0.44	6.11 ± 0.30 *	8.62 ± 0.04	10.80 ± 0.24 *
In total	39.18	57.28	30.02	36.96	27.27	34.55	22.03	27.80
Bitter amino acids								
Val	8.21 ± 0.20	9.86 ± 0.34 *	6.65 ± 0.08	8.27 ± 0.24 *	6.06 ± 1.06	8.98 ± 0.30 *	2.40 ± 0.30	3.24 ± 0.14 *
Met	0.26 ± 0.03	0.30 ± 0.01 *	4.11 ± 0.31	0.28 ± 0.19 *	5.23 ± 0.03	0.91 ± 0.10 *	3.27 ± 0.19	0.69 ± 0.06 *
Ile	4.65 ± 0.01	6.08 ± 0.14 *	3.32 ± 0.13	4.47 ± 0.05 *	4.11 ± 0.10	5.27 ± 0.06 *	2.00 ± 0.01	2.89 ± 0.10 *
Leu	7.14 ± 0.01	8.86 ± 0.26 *	5.61 ± 0.05	6.65 ± 0.06*	6.72 ± 0.07	8.00 ± 0.11 *	3.32 ± 0.03	4.44 ± 0.19 *
Tyr	2.35 ± 0.10	3.01 ± 0.05 *	1.99 ± 0.03	2.67 ± 0.09 *	2.29 ± 0.02	3.02 ± 0.02 *	1.39 ± 0.49	1.71 ± 0.04
Phe	3.98 ± 0.05	5.18 ± 0.13 *	2.64 ± 0.02	3.20 ± 0.08 *	3.05 ± 0.05	3.59 ± 0.04 *	1.46 ± 0.03	1.93 ± 0.15 *
His	2.98 ± 0.05	3.97 ± 0.08 *	2.49 ± 0.06	3.17 ± 0.03 *	2.88 ± 0.06	3.52 ± 0.10 *	1.29 ± 0.03	1.94 ± 0.05 *
Arg	8.98 ± 0.11	12.40 ± 0.05 *	2.65 ± 0.12	9.92 ± 0.47 *	1.59 ± 0.11	11.02 ± 0.22 *	1.24 ± 0.04	6.60 ± 0.06 *
In total	38.54	49.66	29.46	38.62	31.93	44.31	16.36	23.44
Odorless amino acids								
Cys	0.21 ± 0.02	0.23 ± 0.04 *	2.41 ± 0.63	0.34 ± 0.05 *	0.65 ± 0.28	0.37 ± 0.02	-	-
In total	122.44	167.24	99.54	122.99	105.05	138.13	62.27	84.72

Data are shown as means ± standard deviation (n = 3).

Means with * within a column are significantly different compared with NF ($p < 0.05$).

Table S5 Detailed information and contents of main VCs in NF

Number	Compounds	CAS	RI	The contents of VCs in NF (μg/100g)			
				10D	30D	60D	90D
**Acids**							
1	4-Propylbenzoic acid	2438-05-3	1462	ND	ND	ND	279.39 ± 33.22
2	Hexadecanoic acid	57-10-3	1968	ND	ND	ND	150.61 ± 0.15
3	Acetic acid	64-19-7	576	118.58 ± 29.63	96.33 ± 47.53	2284.17 ± 416.32	1756.49 ± 457.75
4	p-tert-Butylbenzoic acid	98-73-7	1477	ND	ND	123.53 ± 12.94	193.31 ± 14.38
**Alcohols**							
5	trans-Farnesol	106-28-5	1710	ND	ND	55.49 ± 8.87	ND
6	D-Nerolidol	142-50-7	1564	23.14 ± 0.71	ND	193.63 ± 33.45	ND
7	cis-4-Thujanol	15537-55-0	1041	ND	ND	ND	222.69 ± 15.82
8	α-muurolol	19435-97-3	1580	ND	ND	39.93 ± 1.33	ND
9	(2Z,6Z)-2,6-Dimethyl-2,6-octadiene-1,8-diol	26489-17-8	1471	ND	ND	ND	177.91 ± 9.20
10	(Z)-9-Tetradecen-1-ol	35153-15-2	1664	ND	7.52 ± 1.52	74.70 ± 7.75	57.97 ± 9.32
11	(Z,E)-Farnesol	3790-71-4	1710	ND	ND	2810.94 ± 411.65	2839.06 ± 185.10
12	Carotol	465-28-1	1593	ND	ND	359.91 ± 117.50	ND
13	α-Bisabolol	515-69-5	1625	ND	14.00 ± 3.63	ND	ND
14	Myrcenol	543-39-5	1064	ND	3.73 ± 0.03	100.92 ± 7.74	ND
15	3-Tetradecyn-1-ol	55182-74-6	1673	ND	137.07 ± 5.06	ND	ND
16	Viridiflorol	552-02-3	1530	ND	0.99 ± 0.20	ND	ND
17	Ocimenol	5986-38-9	1082	ND	ND	86.09 ± 3.81	ND
18	Linalool oxide	5989-33-3	1164	ND	ND	33.93 ± 2.88	ND
19	(E)-β-Terpineol	7299-40-3	1158	ND	85.32 ± 22.58	2079.20 ± 254.75	ND

20	Linalool	78-70-6	1082	158.20 ± 26.66	169.08 ± 45.76	3615.89 ± 415.19	2046.01 ± 230.32
**Aldehydes**							
21	(E,E)-2,4-Hexadienal	142-83-6	822	3.3 ± 0.20	ND	ND	ND
22	(E,E)-2,4-Heptadienal	4313-03-5	921	ND	ND	49.20 ± 10.65	ND
23	Benzaldehyde	100-52-7	982	ND	2.90 ± 1.87	144.96 ± 33.51	67.74 ± 8.76
24	Benzenepropanal	104-53-0	1181	ND	ND	17.51 ± 3.05	ND
25	Anisaldehyde	123-11-5	1171	79.55 ± 24.17	33.13 ± 7.18	1334.49 ± 137.52	4295.39 ± 1478.80
26	Tetradecanal	124-25-4	1601	ND	0.67 ± 0.10	13.66 ± 1.71	25.55 ± 3.23
27	2-Methoxy cinnamaldehyde	1504-74-1	1378	ND	ND	ND	309.65 ± 55.37
28	α-Sinensal	17909-77-2	1646	ND	ND	60.84 ± 7.19	33.80 ± 5.73
29	Methyl coniferyl aldehyde	4497-40-9	1567	ND	ND	ND	22.51 ± 1.66
30	(E)-2-Undecenal	53448-07-0	1311	ND	ND	ND	30.19 ± 14.15
31	2-Hydroxy-4-methoxybenzaldehyde	673-22-3	1392	ND	1.97 ± 0.11	91.33 ± 7.52	ND
**Heterocycles**							
32	Durenol	527-35-5	1354	ND	1.16 ± 0.13	52.29 ± 0.82	ND
33	1-(2-Methoxyphenyl)-2-nitroethene	3316-24-3	1472	ND	ND	171.94 ± 6.20	185.03 ± 139.02
34	1,2-Epoxyhexadecane	7320-37-8	1702	ND	ND	ND	26.74 ± 3.50
35	N-Methyl-1-naphthylamine	2216-68-4	1543	ND	ND	91.74 ± 25.37	137.61 ± 15.15
36	4-Acetoxy-1-decene	2833-33-2	1307	ND	0.76 ± 0.14	ND	ND
37	2-Methoxy-diphenylmethane	883-90-9	1656	ND	ND	ND	110.24 ± 0.45
**Ethers**							
38	Anethole	104-46-1	1190	112.32 ± 18.77	160.18 ± 22.84	3939.68 ± 418.30	3611.62 ± 452.71

39	Nerol oxide	1786-08-9	1125	ND	ND	27.82 ± 0.69	54.10 ± 16.13
40	1,4-Cineol	470-67-7	1012	ND	35.04 ± 2.70	ND	ND
**Esters**							
41	β-Terpinylacetate	10198-23-9	1348	ND	28.04 ± 3.21	421.70 ± 2.90	512.02 ± 234.84
42	Cinnamyl acetate	103-54-8	1367	4.48 ± 0.75	ND	ND	ND
43	Cinnamyl propionate	103-56-0	1466	ND	15.24 ± 0.96	538.48 ± 64.60	920.54 ± 85.88
44	Hexadecanoic acid, methyl ester	112-39-0	1878	ND	ND	ND	38.53 ± 7.84
45	Linalyl formate	115-99-1	1270	491.13 ± 31.34	348.98 ± 1.19	3223.89 ± 291.55	2525.13 ± 453.62
46	Benzenepropyl acetate	122-72-5	1359	ND	6.59 ± 0.22	188.56 ± 12.39	256.07 ± 40.49
47	Nerol acetate	141-12-8	1352	7.01 ± 0.27	11.11 ± 0.75	231.54 ± 36.22	145.64 ± 34.77
48	Ethyl p-methoxycinnamate	1929-30-2	1556	221.04 ± 74.98	194.77 ± 13.96	2503.88 ± 406.61	11429.58 ± 110.43
49	Methyl 3-methoxybenzoate	5368-81-0	1249	ND	ND	ND	241.43 ± 20.93
50	Decyl chloroformate	55488-51-2	1458	ND	ND	12.29 ± 1.01	ND
51	Cyclotene butyrate	68227-51-0	1398	ND	4.32 ± 2.22	ND	ND
52	n-Propyl cinnamate	7778-83-8	1466	ND	209.34 ± 8.71	5040.15 ± 463.11	8239.09 ± 1457.51
53	α-Terpinyl acetate	80-26-2	1333	40.05 ± 1.96	1.04 ± 0.11	ND	34.07 ± 12.63
54	Coumarin	91-64-5	1374	ND	ND	ND	126.43 ± 49.26
55	Eugenol acetate	93-28-7	1552	ND	ND	6.99 ± 4.51	ND
**Ketones**							
56	4-Acetylanisole	100-06-1	1218	ND	8.02 ± 1.62	ND	123.56 ± 54.88
57	p-Acetonylanisole	122-84-9	1318	ND	ND	156.50 ± 25.10	255.67 ± 78.82
58	Dihydro-β-ionone	17283-81-7	1449	ND	1.88 ± 0.38	ND	ND
59	2-Pentadecanone	2345-28-0	1648	ND	ND	5.56 ± 3.01	11.01 ± 2.82

60	2-Bromo-3'-methoxyacetophenone	5000-65-7	1514	ND	0.63 ± 0.28	46.45 ± 5.01	48.74 ± 0.39
61	Eucarvone	503-93-5	1199	ND	ND	14.94 ± 0.55	ND
**Terpenoids**							
62	Valencene	4630-07-3	1474	76.27 ± 7.81	47.95 ± 3.06	ND	272.66 ± 8.80
63	α-Muurolen	10208-80-7	1440	594.12 ± 31.75	310.67 ± 15.14	29.00 ± 7.05	1385.98 ± 128.80
64	Cedrene	11028-42-5	1398	ND	18.69 ± 1.42	241.45 ± 31.69	115.85 ± 7.01
65	Isocaryophyllene	118-65-0	1494	ND	2.96 ± 0.17	ND	ND
66	Isosafrole	120-58-1	1668	ND	1.54 ± 0.12	42.67 ± 2.12	56.34 ± 5.66
67	β-Myrcene	123-35-3	958	18.01 ± 0.38	8.92 ± 2.76	432.82 ± 45.09	247.80 ± 18.06
68	(E)-α-Bergamotene	13474-59-4	1430	ND	24.11 ± 0.17	305.36 ± 50.09	174.84 ± 0.33
69	β-Ocimene	13877-91-3	976	9.05 ± 0.20	10.53 ± 1.99	223.22 ± 2.54	146.94 ± 29.37
70	α-Bergamotene	17699-05-7	1430	135.99 ± 12.82	ND	ND	ND
71	α-Cubebene	17699-14-8	1344	414.02 ± 55.46	246.42 ± 44.26	2762.87 ± 421.72	903.29 ± 160.42
72	(E)-β-Famesene	18794-84-8	1440	55.12 ± 2.64	ND	73.25 ± 11.74	114.23 ± 6.05
73	Humulene epoxide II	19888-34-7	1592	ND	ND	15.86 ± 4.51	ND
74	β-Sesquiphellandrene	20307-83-9	1446	ND	31.23 ± 3.85	201.82 ± 8.57	ND
75	α-Calacorene	21391-99-1	1547	12.83 ± 2.39	8.59 ± 2.15	20.41 ± 3.70	129.56 ± 20.95
76	Germacrene D	23986-74-5	1515	244.66 ± 2.65	144.85 ± 12.91	223.22 ± 4.40	731.65 ± 117.48
77	α-Bisabolene	29837-07-8	1518	ND	ND	ND	31.48 ± 11.18
78	β-elemene	33880-83-0	1398	ND	9.69 ± 1.02	91.38 ± 5.49	58.91 ± 6.70
79	(+)-Sativen	3650-28-0	1339	ND	11.72 ± 9.37	13.00 ± 2.12	ND
80	α-Bulnesene	3691-11-0	1490	ND	ND	60.00 ± 11.20	63.04 ± 11.32
81	Fenchene	471-84-1	943	ND	ND	112.10 ± 1.83	ND
82	α-Selinene	473-13-2	985	ND	1.88 ± 0.66	ND	ND
83	Δ-Cadinene	483-76-1	1469	ND	ND	447.30 ± 27.19	ND

84	L-calamenene	483-77-2	1537	42.62 ± 2.71	46.61 ± 0.54	275.90 ± 31.89	269.05 ± 16.09
85	Aromandendrene	489-39-4	1386	40.85 ± 6.33	46.13 ± 6.89	366.49 ± 19.88	154.53 ± 6.82
86	β-Bisabolene	495-61-4	1500	25.77 ± 0.86	37.90 ± 15.39	212.02 ± 19.98	320.90 ± 45.11
87	γ-Patchoulene	508-55-4	1398	ND	15.07 ± 1.39	ND	ND
88	Terpinolene	586-62-9	1052	ND	0.21 ± 0.02	40.52 ± 8.57	12.00 ± 1.46
89	D-Limonene	5989-27-5	1018	57.09 ± 5.80	13.77 ± 5.88	1038.64 ± 100.28	ND
90	(-)-Aristolene	6831-16-9	1403	ND	1.94 ± 0.24	ND	ND
91	γ-Terpinene	99-85-4	998	9.41 ± 2.02	4.23 ± 0.52	83.16 ± 13.03	ND
**Hydrocarbons**							
92	1-Pentadecene	13360-61-7	1502	ND	20.36 ± 0.74	224.66 ± 17.12	ND
93	1,3,8-p-Menthatriene	18368-95-1	1029	ND	0.88 ± 0.00	27.96 ± 2.72	7.43 ± 0.87
95	β-Methylisoallylbenzene	768-49-0	1077	ND	ND	ND	35.07 ± 6.01
96	Hexadecane	544-76-3	1612	5.87 ± 0.74	3.45 ± 0.79	44.36 ± 12.13	63.67 ± 9.90
97	2-Methyl-6-propyldodecane	55045-08-4	1483	ND	ND	ND	4170.21 ± 59.74
98	Pentadecane	629-62-9	1512	1172.55 ± 58.21	807.37 ± 74.39	12291.40 ± 1507.37	ND
99	Heptadecane	629-78-7	1711	40.86 ± 3.79	24.00 ± 7.11	265.41 ± 41.80	316.39 ± 16.54
100	8-Hexadecyne	19781-86-3	1629	12.65 ± 0.51	ND	ND	ND

Data are shown as means ± standard deviation (n = 3).
ND means not detected

Table S6. Detailed information and contents of main VCs in BMF

Number	Compounds	CAS	RI	The contents of VCs in BMF (μg/100g)			
				10D	30D	60D	90D
**Acids**				ND	ND	ND	ND
1	Hexadecanoic acid	57-10-3	1968	ND	ND	ND	80.81 ± 9.46
2	Acetic acid	64-19-7	576	27.15 ± 5.76	38.24 ± 4.49	1101.79 ± 67.37	2580.26 ± 432.93
3	p-tert-Butylbenzoic acid	98-73-7	1477	ND	ND	ND	190.95 ± 32.33
**Alcohols**							
4	β-Terpineol	138-87-4	1158	ND	59.36 ± 5.54	ND	ND
5	D-Nerolidol	142-50-7	1564	8.19 ± 6.10	5.99 ± 1.52	167.43 ± 23.62	ND
6	cis-4-Thujanol	15537-55-0	1041	ND	1.45 ± 0.06	717.07 ± 248.01	414.41 ± 88.30
7	Cubenol	21284-22-0	1580	ND	5.33 ± 0.23	ND	ND
8	(Z)-9-Tetradecen-1-ol	35153-15-2	1664	ND	4.24 ± 0.32	109.08 ± 8.57	88.05 ± 15.08
9	(Z,E)-Farnesol	3790-71-4	1710	ND	50.01 ± 7.07	3617.19 ± 527.25	3077.64 ± 823.55
10	α-Cadinol	481-34-5	1580	ND	ND	117.61 ± 9.99	ND
11	α-Bisabolol	515-69-5	1625	ND	6.87 ± 1.12	347.34 ± 34.20	ND
12	Myrcenol	543-39-5	1064	ND	2.65 ± 0.92	86.61 ± 1.01	76.17 ± 10.82
13	Viridiflorol	552-02-3	1530	ND	0.58 ± 0.12	38.09 ± 3.01	ND
14	Terpinen-4-ol	562-74-3	1137	61.60 ± 14.58	ND	1773.93 ± 375.75	1502.45 ± 386.08
15	γ-Terpineol	586-81-2	1191	ND	ND	ND	756.25 ± 212.99
16	Ocimenol	5986-38-9	1082	ND	ND	74.56 ± 5.36	ND
17	Nerolidol	7212-44-4	1564	ND	ND	297.89 ± 15.14	ND
18	2,4-Undecadienol	77657-78-4	1373	ND	ND	30.18 ± 9.94	ND
19	Linalool	78-70-6	1082	109.61 ± 16.08	92.59 ± 6.81	3269.20 ± 932.74	4355.95 ± 585.54
**Aldehydes**							

20	(E,E)-2,4-Hexadienal	142-83-6	822	2.15 ± 0.03	ND	ND	ND
21	(E,E)-2,4-Heptadienal	4313-03-5	921	ND	1.28 ± 0.31	34.44 ± 4.09	42.29 ± 5.98
22	Benzaldehyde	100-52-7	982	0.96 ± 0.16	0.86 ± 0.06	70.72 ± 18.80	95.14 ± 20.68
23	Benzenepropanal	104-53-0	1181	3.25 ± 0.48	4.13 ± 1.05	56.39 ± 6.83	83.76 ± 11.15
24	Neral	106-26-3	1174	ND	ND	12.31 ± 3.32	ND
25	Anisaldehyde	123-11-5	1171	19.85 ± 1.97	17.49 ± 0.94	1180.24 ± 117.02	4380.91 ± 156.99
26	Tetradecanal	124-25-4	1601	ND	0.23 ± 0.02	17.13 ± 0.04	25.90 ± 6.83
27	2-Methoxy cinnamaldehyde	1504-74-1	1378	16.36 ± 1.00	ND	236.57 ± 14.56	329.93 ± 33.74
28	α-Sinensal	17909-77-2	1646	ND	ND	27.07 ± 3.20	ND
29	2-Hydroxy-4-methoxybenzaldehyde	673-22-3	1392	ND	ND	66.38 ± 19.32	58.34 ± 25.53
30	Furfural	98-01-1	831	ND	ND	ND	161.78 ± 11.44
**Heterocycles**							
31	α-Durenol	4393-05-9	1376	ND	1.79 ± 0.26	ND	ND
32	Durenol	527-35-5	1354	ND	ND	76.43 ± 4.82	ND
33	2-Methoxy-diphenylmethane	883-90-9	1656	ND	ND	100.80 ± 8.83	115.68 ± 16.95
34	p-Anisoyl chloride	100-07-2	1295	ND	ND	76.73 ± 6.66	ND
35	N-Methyl-1-naphthylamine	2216-68-4	1543	ND	2.80 ± 1.12	111.02 ± 3.11	101.33 ± 5.24
**Ethers**							
36	Anethole	104-46-1	1190	114.30 ± 21.37	66.39 ± 2.18	3450.49 ± 1240.81	2679.23 ± 73.35
37	Nerol oxide	1786-08-9	1125	ND	ND	33.00 ± 3.58	72.63 ± 15.05
38	Isoeugenyl methyl ether	93-16-3	1379	ND	ND	122.42 ± 8.62	ND
**Esters**							
39	β-Terpinylacetate	10198-23-9	1348	ND	15.04- ± 4.52	517.95 ± 31.91	434.50 ± 18.31
40	Methyl cinnamate	103-26-4	1267	0.99- ± 0.08	ND	ND	ND

41	Ethyl cinnamate	103-36-6	1367	220.80 ± 15.46	ND	ND	ND
42	Cinnamyl acetate	103-54-8	1367	ND	3.03 ± 0.53	268.11 ± 26.44	439.83 ± 85.54
43	Crotonic acid, o-formylphenyl ester	114636-69-0	1569	ND	ND	159.50 ± 8.25	181.20 ± 35.92
44	Linalyl formate	115-99-1	1270	417.96 ± 24.45	134.74 ± 15.51	3645.34 ± 237.77	1934.83 ± 96.40
45	Benzenepropyl acetate	122-72-5	1359	ND	3.56 ± 0.47	151.29 ± 21.66	273.40 ± 20.71
46	Nerol acetate	141-12-8	1352	5.41 ± 0.32	4.71 ± 0.83	268.66 ± 31.68	181.54 ± 49.13
47	Ethyl p-methoxycinnamate	1929-30-2	1556	149.19 ± 34.01	89.22 ± 5.15	737.38 ± 121.45	11477.28 ± 867.84
48	Decyl chloroformate	55488-51-2	1458	ND	0.63 ± 0.07	ND	ND
49	Cyclotene butyrate	68227-51-0	1398	ND	ND	43.35 ± 7.18	ND
50	2,2,4-Trimethyl-1,3-pentanediol diisobutyrate	6846-50-0	1605	ND	0.66 ± 0.10	ND	ND
51	Methyl 15-methylhexadecanoate	6929-04-0	1914	ND	ND	ND	32.95 ± 4.65
52	Allyl thiocyanate	764-49-8	905	ND	ND	13.67 ± 2.14	ND
53	n-Propyl cinnamate	7778-83-8	1466	ND	121.77 ± 28.27	5508.86 ± 352.46	7202.57 ± 168.63
54	α-Terpinyl acetate	80-26-2	1333	31.51 ± 0.77	ND	24.55 ± 5.41	40.07 ± 5.16
55	Coumarin	91-64-5	1374	ND	ND	ND	118.24 ± 12.08
**Ketones**							
56	4-Acetylanisole	100-06-1	1218	ND	ND	ND	115.79 ± 11.05
57	p-Methoxypropiophenone	121-97-1	1318	ND	2.84 ± 0.04	ND	150.55 ± 14.13
58	p-Acetonylanisole	122-84-9	1318	ND	3.76 ± 1.21	160.74 ± 19.54	225.99 ± 14.57
59	9-Methyl-5-methylene-8-decen-2-one	130876-97-0	1302	ND	1.15 ± 0.26	77.84 ± 18.24	137.98 ± 19.69
60	2-Pentadecanone	2345-28-0	1648	ND	ND	9.73 ± 4.01	ND
61	Geranyl acetone	3796-70-1	1420	ND	ND	105.57 ± 42.27	ND

62	2-Bromo-3'-methoxyacetophenone	5000-65-7	1514	ND	ND	39.74 ± 7.60	264.85 ± 29.21
63	Eucarvone	503-93-5	1199	ND	2.76 ± 1.00	15.93 ± 2.59	ND
**Terpenoids**							
64	Valencene	4630-07-3	1474	51.09 ± 9.61	15.60 ± 5.02	ND	ND
65	α-Muurolen	10208-80-7	1440	320.92 ± 72.04	83.40 ± 39.85	2961.93 ± 377.73	2595.56 ± 348.38
66	Cedrene	11028-42-5	1398	ND	7.83 ± 0.01	209.45 ± 26.10	138.90 ± 11.64
67	Isosafrole	120-58-1	1668	ND	1.11 ± 0.36	50.87 ± 5.62	66.02 ± 5.42
68	β-Myrcene	123-35-3	958	11.70 ± 1.17	6.51 ± 1.25	427.87 ± 41.20	251.84 ± 80.05
69	(E)-α-Bergamotene	13474-59-4	1430	73.13 ± 18.45	12.16 ± 0.44	279.19 ± 37.04	259.55 ± 81.16
70	β-Cubebene	13744-15-5	1339	ND	ND	148.28 ± 3.88	ND
71	β-Ocimene	13877-91-3	976	5.08 ± 0.43	4.76 ± 0.64	259.81 ± 24.74	205.33 ± 78.18
72	β-Himachalene	1461-03-6	1528	ND	16.54 ± 2.90	404.72 ± 68.73	413.30 ± 121.87
73	α-Bergamotene	17699-05-7	1430	ND	22.02 ± 2.67	533.46 ± 3.89	320.23 ± 34.58
74	α-Cubebene	17699-14-8	1344	294.11 ± 74.10	93.86 ± 3.65	2804.45 ± 327.07	1556.55 ± 144.19
75	(E)-β-Famesene	18794-84-8	1440	26.96 ± 7.53	ND	ND	ND
76	Humulene epoxide II	19888-34-7	1592	ND	ND	104.82 ± 2.86	18.60 ± 2.69
77	β-Sesquiphellandrene	20307-83-9	1446	ND	3.55 ± 0.35	258.77 ± 5.51	170.05 ± 26.44
78	α-Calacorene	21391-99-1	1547	5.78 ± 1.80	ND	ND	9.57 ± 2.22
79	Germacrene D	23986-74-5	1515	99.71 ± 44.84	81.76 ± 8.97	269.22 ± 1.52	1259.32 ± 63.52
80	α-Bisabolene	29837-07-8	1518	ND	ND	ND	40.61 ± 5.39
81	γ-Elemene	29873-99-2	1431	ND	1.46 ± 0.07	ND	ND
82	β-elemene	33880-83-0	1398	11.10 ± 2.30	3.16 ± 1.03	97.57 ± 13.44	104.21 ± 40.85
83	(+)-Sativen	3650-28-0	1339	ND	7.32 ± 2.17	160.53 ± 37.27	ND
84	α-Bulnesene	3691-11-0	1490	4.66 ± 0.72	ND	ND	71.52 ± 29.85
85	Fenchene	471-84-1	943	ND	ND	134.79 ± 51.46	ND

86	α-Selinene	473-13-2	985	ND	2.56 ± 1.10	53.39 ± 3.40	ND
87	Δ-Cadinene	483-76-1	1469	ND	ND	470.66 ± 88.54	486.90 ± 177.38
88	L-calamenene	483-77-2	1537	18.91 ± 4.71	26.19 ± 5.64	320.41 ± 5.21	335.59 ± 32.35
89	Aromandendrene	489-39-4	1386	23.47 ± 4.25	15.53 ± 4.69	315.01 ± 30.94	381.88 ± 30.14
90	β-Bisabolene	495-61-4	1500	17.10 ± 4.60	14.27 ± 0.93	213.51 ± 7.54	400.75 ± 99.31
91	Pseudolimonene	499-97-8	1013	12.93 ± 2.57	1.14 ± 0.28	ND	ND
92	γ-Patchoulene	508-55-4	1398	ND	ND	153.59 ± 14.83	ND
93	Terpinolene	586-62-9	1052	ND	ND	61.68 ± 1.02	9.04 ± 8.44
94	D-Limonene	5989-27-5	1018	46.32 ± 18.50	17.89 ± 0.71	ND	ND
95	(-)-Aristolene	6831-16-9	1403	ND	4.19 ± 2.88	ND	ND
96	(+)-α-pinene	7785-70-8	948	1.85 ± 0.20	ND	ND	ND
97	γ-Terpinene	99-85-4	998	ND	ND	68.46 ± 32.00	ND
**Hydrocarbons**							
98	1-Pentadecene	13360-61-7	1502	ND	8.27 ± 1.09	240.85 ± 25.95	247.45 ± 67.37
99	1,3,8-p-Menthatriene	18368-95-1	1029	0.87 ± 0.01	ND	19.99 ± 0.66	ND
100	2-Methyl-5-oxo-1-cyclopenten-1-yl butyrate	62338-57-2	1013	ND	ND	ND	8.31 ± 4.60
101	Dihydrosafrole	94-58-6	1337	ND	ND	66.65 ± 12.77	ND
102	Hexadecane	544-76-3	1612	2.81 ± 0.50	1.50 ± 0.24	50.96 ± 9.93	ND
103	2-Methyl-6-propyldodecane	55045-08-4	1483	ND	ND	ND	6510.08 ± 542.21
104	Pentadecane	629-62-9	1512	1367.34 ± 337.47	411.98 ± 158.37	12996.68 ± 297.43	ND
105	Heptadecane	629-78-7	1711	21.68 ± 5.54	7.13 ± 0.81	443.81 ± 55.16	418.38 ± 140.17
106	8-Hexadecyne	19781-86-3	1629	6.86 ± 1.42	ND	ND	ND

Data are shown as means ± standard deviation (n = 3).
ND means not detected

Table S7. Ordinal list of metabolites in S-plot

No.	Compounds	No.	Compounds	No.	Compounds	No.	Compounds	No.	Compounds	No.	Compounds
1	4-Propylbenzoic acid	26	2,4-Undecadienol	51	1,4-Cineol	76	9-Methyl-5-methylene-8-decen-2-one	101	β-elemene	126	8-Hexadecyne
2	Hexadecanoic acid	27	Linalool	52	Isoeugenyl methyl ether	77	Dihydro-β-ionone	102	(+)-Sativen	127	Asp
3	Acetic acid	28	(E,E)-2,4-Hexadienal	53	β-Terpinylacetate	78	2-Pentadecanone	103	α-Bulnesene	128	Glu
4	p-tert-Butylbenzoic acid	29	(E,E)-2,4-Heptadienal	54	Methyl cinnamate	79	Geranyl acetone	104	Fenchene	129	Thr
5	trans-Farnesol	30	Benzaldehyde	55	Cinnamyl acetate	80	2-Bromo-3'-methoxyacetophenone	105	α-Selinene	130	Ser
6	β-Terpineol	31	Benzenepropanal	56	Cinnamyl propionate	81	Eucarvone	106	Δ-Cadinene	131	Gly
7	D-Nerolidol	32	Neral	57	Crotonic acid, o-formylphenyl ester	82	Valencene	107	L-calamenene	132	Ala
8	cis-4-Thujanol	33	Anisaldehyde	58	Hexadecanoic acid, methyl ester	83	α-Muurolen	108	Aromandendrene	133	Lys
9	α-muurolol	34	Tetradecanal	59	Linalyl formate	84	Cedrene	109	β-Bisabolene	134	Pro
10	(2Z,6Z)-2,6-Dimethyl-2,6-octadiene-1,8-diol	35	2-Methoxy cinnamaldehyde	60	Benzenepropyl acetate	85	Isocaryophyllene	110	Pseudolimonene	135	Val
11	Cubenol	36	α-Sinensal	61	Nerol acetate	86	Isosafrole	111	γ-Patchoulene	136	Met
12	(Z)-9-Tetradecen-1-ol	37	(E)-2-Undecenal	62	Ethyl p-methoxycinnamate	87	β-Myrcene	112	Terpinolene	137	Ile
13	(Z,E)-Farnesol	38	methyl coniferyl aldehyde	63	Methyl 3-methoxybenzoate	88	(E)-α-Bergamotene	113	D-Limonene	138	Leu
14	Carotol	39	2-Hydroxy-4-methoxybenzaldehyde	64	Decyl chloroformate	89	β-Cubebene	114	(-)-Aristolene	139	Tyr
15	α-Cadinol	40	Furfural	65	Cyclotene butyrate	90	β-Ocimene	115	(+)-α-pinene	140	Phe

16	α-Bisabolol	41	α-Durenol	66	2,2,4-Trimethyl-1,3-pentanediol diisobutyrate	91	β-Himachalene	116	γ-Terpinene	141	His
17	Myrcenol	42	Durenol	67	Methyl 15-methylhexadecanoate	92	α-Bergamotene	117	2-Methyl-5-oxo-1-cyclopenten-1-yl butyrate	142	Arg
18	3-Tetradecyn-1-ol	43	2-Methoxy-diphenylmethane	68	Allyl thiocyanate	93	α-Cubebene	118	Dihydrosafrole	143	(Cys)$_2$
19	Viridiflorol	44	1-(2-Methoxyphenyl)-2-nitroethene	69	n-Propyl cinnamate	94	(E)-β-Famesene	119	β-Methylisoallylbenzene	144	Oxalic acid
20	Terpinen-4-ol	45	1,2-Epoxyhexadecane	70	α-Terpinyl acetate	95	Humulene epoxide II	120	1-Pentadecene	145	Tartaric acid
21	γ-Terpineol	46	N-Methyl-1-naphthylamine	71	Coumarin	96	β-Sesquiphellandrene	121	1,3,8-p-Menthatriene	146	Malic acid
22	Ocimenol	47	p-Anisoyl chloride	72	Eugenol acetate	97	α-Calacorene	122	Hexadecane	147	Lactic acid
23	Linalool oxide	48	4-Acetoxy-1-decene	73	4-Acetylanisole	98	Germacrene D	123	2-Methyl-6-propyldodecane	148	Acetic acid
24	Nerolidol	49	Anethole	74	p-Methoxypropiophenone	99	α-Bisabolene	124	Pentadecane	149	Citric acid
25	(E)-β-Terpineol	50	Nerol oxide	75	p-Acetonylanisole	100	γ-Elemene	125	Heptadecane	150	Succinic acid

Table S8 Differential metabolites between NF and BMF

	Compounds	No. [a]	VIP
**FAAs**	Glu	128	1.59
	Phe	140	1.30
	Asp	127	1.28
	Pro	134	1.27
	Arg	142	1.23
	Ala	132	1.20
	Met	136	1.14
	Val	135	1.10
**OAs**	Lactic acid	147	1.19
	Malic acid	146	1.17
	Tartaric acid	145	1.05
**VCs**	Terpinen-4-ol	20	3.48
	α-Muurolen	9	3.25
	n-Propyl cinnamate	69	2.47
	2-Methyl-6-propyldodecane	123	2.43
	(E)-β-Terpineol	25	2.43
	Cinnamyl propionate	56	2.25
	Linalool	27	2.07
	Anethole	49	1.99
	(Z,E)-Farnesol	13	1.79
	β-Himachalene	91	1.70
	D-Limonene	113	1.67
	cis-4-Thujanol	8	1.61
	Linalyl formate	59	1.59
	α-Bergamotene	92	1.58
	Cinnamyl acetate	55	1.54
	γ-Terpineol	21	1.45
	Acetic acid	3	1.42
	Crotonic acid, o-formylphenyl ester	57	1.09

δ-Cadinene	106	1.06
Valencene	82	1.02

a: The numbers are consistent with Table S7
